# Spatially Resolved 2D Laser Writing on Graphene Using Diazonium Salts

**DOI:** 10.1002/chem.202502468

**Published:** 2025-09-13

**Authors:** Johanna Krüger, Tamara Nagel, Bowen Yang, Frank Hauke, Andreas Hirsch

**Affiliations:** ^1^ Department of Chemistry and Pharmacy & Center of Advanced Materials and Processes (ZMP) Friedrich‐Alexander University of Erlangen‐Nürnberg (FAU) Nikolaus‐Fiebiger‐Straße 10 91058 Erlangen Germany

**Keywords:** covalent patterning, diazonium salts, graphene, laser writing, Raman spectroscopy

## Abstract

We present the first investigation of precise laser‐guided patterning, functionalization, defunctionalization, and refunctionalization of single‐layer graphene (SLG) with three different aryl diazonium salts. Using scanning Raman microscopy (SRM) we confirmed the spatially resolved nature of the functionalization. We systematically investigated the influence of key laser parameters (power, irradiation time, and wavelength) and the electronic effects of the substituents on both the functionalization efficiency and integrity of graphene. Our solid‐phase approach unequivocally demonstrates that laser‐triggered covalent attachment can achieve high functionalization levels comparable to solution‐based methods. We also unveil a novel laser‐induced defunctionalization pathway at high laser powers and prolonged irradiation times, which allows for the precise, patterned functional group “erasure” – a significant advantage over nonselective thermal annealing. Furthermore, we successfully demonstrate the successful readdressing and refunctionalization of these laser “erased” areas, enabling a complete “write‐erase‐rewrite” cycle. This work establishes a robust and highly adaptable platform for creating complex, mixed‐functionalized graphene architectures with micrometer precision.

## Graphical abstract

A comprehensive investigation of laser‐induced covalent patterning, defunctionalization, and refunctionalization of single‐layer graphene with aryl diazonium salts is reported. Scanning Raman microscopy verifies spatial control, while systematic variation of laser parameters and substituent effects highlights the versatility of the method. The demonstrated “write‐erase‐rewrite” capability establishes a robust platform for micrometer‐scale fabrication of complex, functionalized graphene architectures.

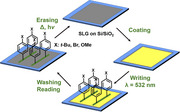

## 1 Introduction

Diazonium salts are among the most effective reagents for the chemical functionalization of graphene. Strano et al. have demonstrated the successful introduction of aryl groups onto single‐layer graphene (SLG) and multi‐layered graphene in solution and on substrates.^[^ ^1^, ^2^ ^]^ This process involves an electron transfer reaction that generates aryl radicals, which subsequently react with the graphene lattice.^[^ ^3^ ^]^ The versatile chemistry of aryl diazonium salts offers significant advantages for tailoring graphene properties.^[^ ^4^, ^5^, ^6^ ^]^ These compounds are easily synthesized from commercially available aryl amines^[^ ^7^, ^8^ ^]^ and are widely used in organic synthesis and surface functionalization, including applications on carbon electrodes, metals, semiconductors, and carbon nanotubes.^[^ ^6^, ^9^, ^10^, ^11^, ^12^, ^13^ ^]^

However, reductive functionalization methods involving highly reactive aryl radicals lack precise control over the quantity and spatial distribution of functional groups. To address this limitation, 2D patterning techniques have been developed. These methods offer precise spatial control, allowing for the modification of graphene's solubility, conductivity, electronic doping, and optical and catalytic activity through the regio‐specific introduction of functional groups.^[^ ^14^, ^15^ ^]^ Electron‐beam lithography (EBL) combined with a poly(methyl methacrylate) (PMMA) mask is a prominent approach for achieving high‐resolution covalent functionalization.^[^ ^14^, ^16^, ^17^ ^]^

In 2020, our group introduced a mask‐free patterning technique known as “laser writing”.^[^ ^18^ ^]^ This approach simplifies the functionalization process by reducing preparation steps, avoiding complex lithographic procedures, and minimizing surface contamination. The approach is based on a photosensitive compound (i.e., dibenzoyl peroxide (DBPO)) deposited on SLG. The spatially resolved 2D functionalization is achieved through a laser‐initiated generation of aryl radicals, which covalently bind exclusively to the laser‐irradiated pathways. Using this method, “writing” of various 2D architectures is possible by adding covalently bound moieties.

Since its initial publication, we have refined the “laser writing” technique by incorporating a washing step between “writing” and “readout” to eliminate any background functionalization from unreacted residues.^[^ ^19^ ^]^ Additionally, we have explored the limits of lateral resolution^[^ ^19^ ^]^ and successfully extended this concept to other compound classes, including hypervalent iodine compounds, silver acetates, and iodonium salts.^[^ ^20^, ^21^, ^22^, ^23^ ^]^ However, until now, the precise 2D patterning of SLG with diazonium salts has remained unexplored.

In this work, we successfully expanded the established “laser writing” approach for DBPO and iodonium salts to incorporate highly versatile diazonium compounds. We developed an optimized patterning protocol for the spatially resolved introduction of three structurally distinct aryl moieties with electron‐withdrawing, electron‐donating, and neutral characteristics. Through systematic parameter optimization – including laser wavelength, laser power, and irradiation time – we gained a deeper understanding of the underlying reaction mechanism, which enabled a precise control over the degree of functionalization. This allowed us to achieve functionalization levels comparable to, or even higher than, established solvent‐based methods. More remarkably, at higher laser powers and prolonged irradiation times, we observed a laser‐induced detachment of the covalently introduced moieties. This allows for the precise, patterned “erasure” of covalently bound functional groups, a significant advantage over traditional thermal annealing, which defunctionalizes graphene uniformly. Furthermore, we demonstrated a complete “write‐erase‐rewrite” cycle of functional entities, confirming that the restored graphene lattice can be readdressed for subsequent covalent functionalization. Scanning Raman microscopy (SRM) was used to confirm spatially resolved functionalization and defunctionalization, with further sample characterization carried out by optical microscopy, atomic force microscopy (AFM), and Kelvin probe force microscopy (KPFM).

## 2 Results and Discussion

**Scheme 1:**
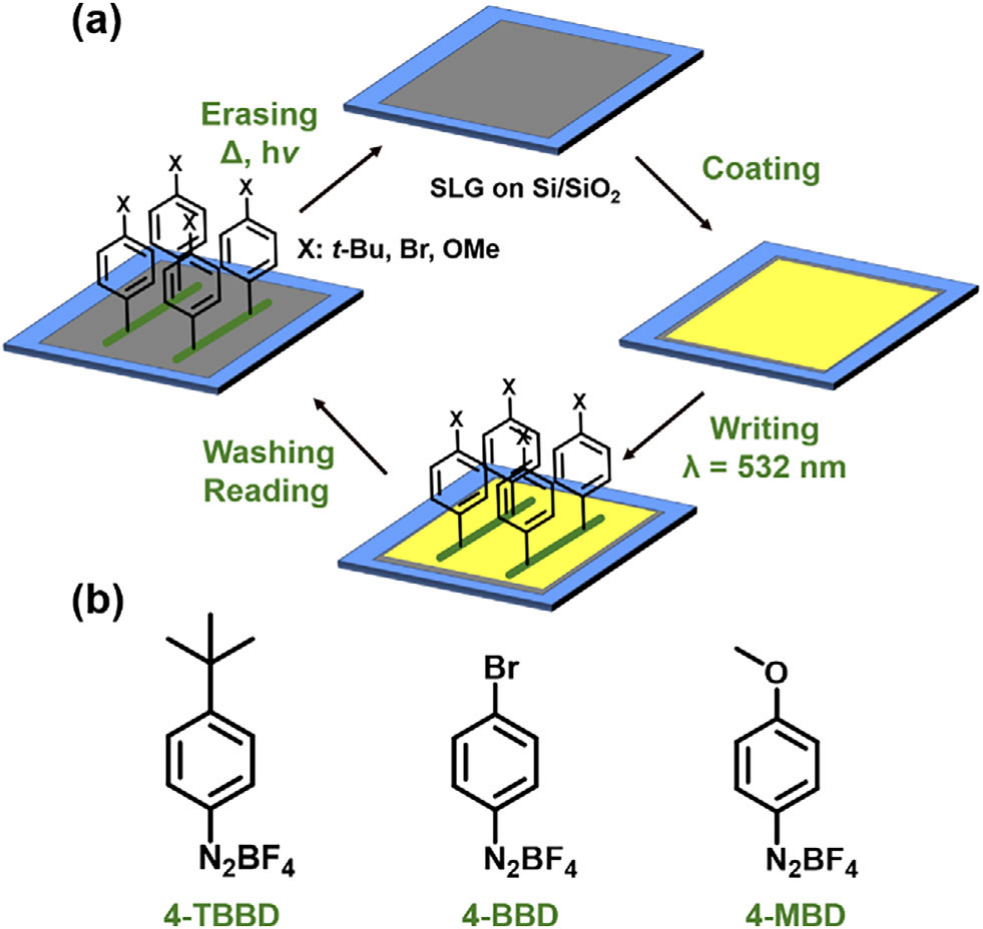
a) Schematic representation of the “laser writing” protocol, including the coating, “writing”, “reading”, and “erasing” steps. b) The diazonium salts used: 4‐*tert*‐butylbenzenediazonium tetrafluoroborate (4‐TBBD), 4‐bromobenzenediazonium tetrafluoroborate (4‐BBD), and 4‐methoxybenzenediazonium tetrafluoroborate (4‐MBD), used to modulate the electronic properties of monolayer graphene.

Scheme 1a outlines the comprehensive “laser writing” protocol for the functionalization of SLG on a Si/SiO_2_ substrate using diazonium salts. The protocol includes the coating, “writing,” “reading,” and “erasing” processes. To investigate the influence of different substituents in the *para* position of the aryl moiety on the covalent functionalization and the respective electronic properties of graphene, three structurally different diazonium salts were selected (Scheme 1b). 4‐*tert*‐Butylbenzenediazonium tetrafluoroborate (4‐TBBD) serves as the model compound and is electrically neutral without pronounced electronic effects. In general, functionalization of the graphene surface with aryl groups bearing different substituents offers the possibility to modulate the electronic properties of graphene. Specifically, 4‐bromobenzenediazonium tetrafluoroborate (4‐BBD) introduces electron‐withdrawing characteristics, while 4‐methoxybenzenediazonium tetrafluoroborate (4‐MBD) can act as an electron‐donating species. This selection underscores the diversity of electronic properties achievable through the chosen diazonium salts.

**Figure 1:**
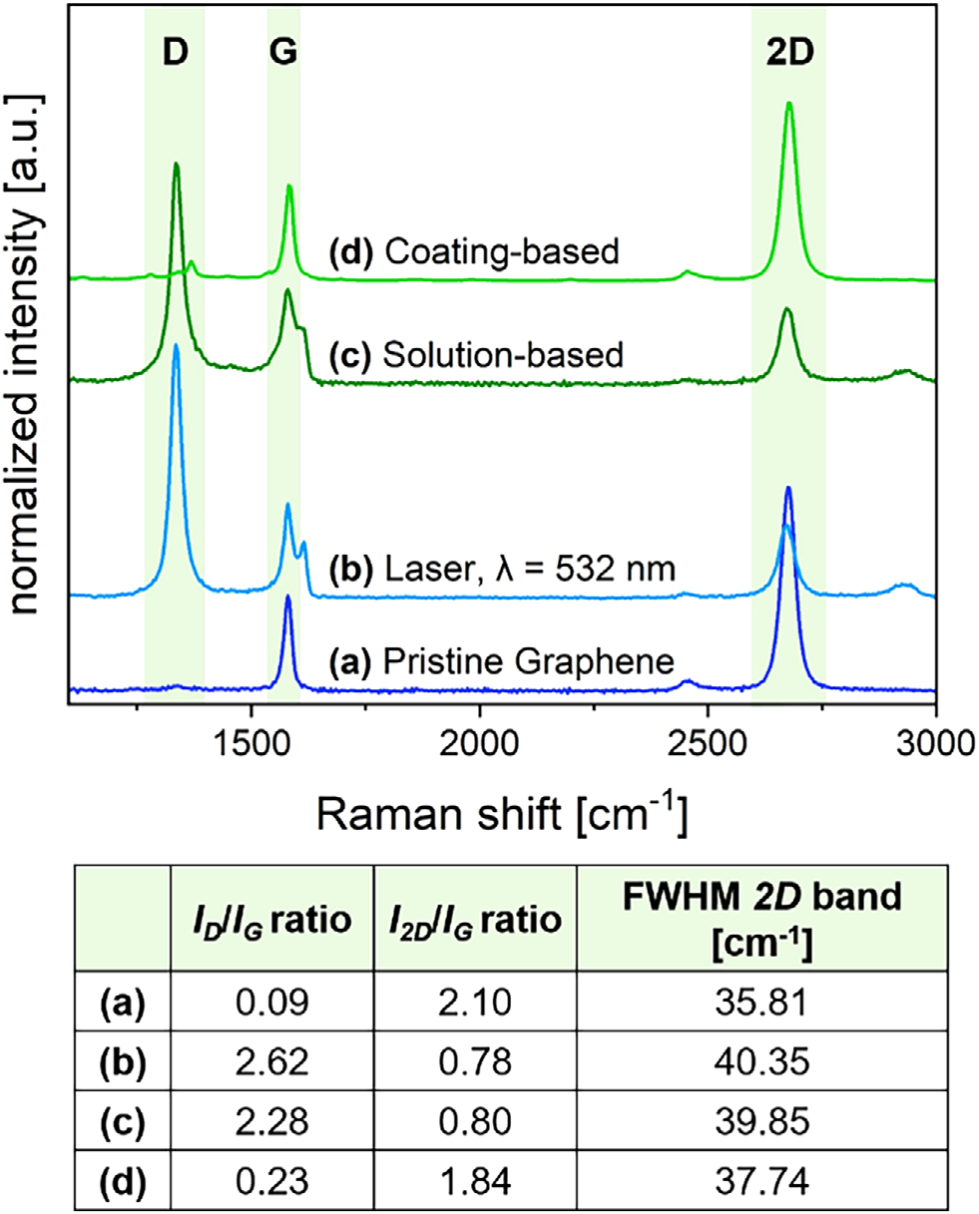
**Top**: a) Weighted and normalized mean Raman spectrum of pristine SLG on Si/SiO_2_ (“readout” parameters: λ_exc_ = 532 nm, 5 mW, 1 s, 0.5 µm step size). b) Normalized single point Raman spectrum after irradiation of a 4‐TBBD film (10^−3^ M in THF) with λ_exc_ = 532 nm, 0.88 mW, 60 s. c) Weighted and normalized mean Raman spectrum of SLG on Si/SiO_2_ after immersing the sample in a solution of 4‐TBBD (1.7⋅10^−2^ M) and SDS (1 wt%) in double distilled (dd) H_2_O for 17 hours and washed with dd H_2_O (“readout” parameters: λ_exc_ = 532 nm, 5 mW, 1 s, 0.5 µm step size). d) Weighted and normalized mean Raman spectrum of SLG on Si/SiO_2_, coated with 4‐TBBD (10^−3^ M in THF), heated at 50 °C for 30 min, and washed with THF (“readout” parameters: λ_exc_ = 532 nm, 4 mW, 0.5 s, 0.5 µm step size). **Bottom**: Summary of the *I_D_*/*I_G_* ratio, *I_2D_*/*I_G_* ratio, and full width at half maximum (FWHM) for Raman spectra a) – d).

At the beginning of the functionalization sequence, CVD graphene was deposited on a Si/SiO_2_ wafer (300 nm SiO_2_) using an optimized wet transfer technique (see Supporting Information for further experimental details). For laser‐induced covalent graphene functionalization, the SLG sample should ideally be uniformly coated with a thin and homogeneous layer of 4‐TBBD. This was achieved by spin‐coating a 10^−3^ M solution of 4‐TBBD in THF on the SLG substrate (optical image of the coating, see Figure S2). Based on previous studies of laser‐triggered graphene functionalization using dibenzoyl peroxides^[^ ^18^, ^19^ ^]^ and λ^3^‐iodanes,^[^ ^23^ ^]^ a green laser (λ_exc_  =  532 nm, 0.88 mW) from our Raman microscope and an irradiation time of 60 s were selected to illuminate the coated graphene sample. The resulting Raman spectrum is shown in Figure 1b. The pronounced increase in the *D* band at around 1340 cm^−1^ intensity unequivocally indicates the successful covalent attachment of intermediately generated aryl radicals. Pristine graphene (Figure 1a) exhibits a prominent *G* band at approximately 1582 cm^−1^ and an indistinguishable *D* band, indicating an intact, defect‐free sp^2^ lattice. Upon covalent functionalization, lattice sp^3^‐hybridized carbon atoms are generated, resulting in an activation of the Raman *D* band.

According to Strano et al.^[^ ^3^ ^]^ the most common reaction mechanism for covalent functionalization with aryl diazonium salts is based on a solid‐phase/solution electron transfer reaction. In this process, graphene donates a delocalized electron to the aryl diazonium cation. This leads to the formation of an aryl radical and the release of a N_2_ molecule. The radical subsequently forms a new bond with a carbon atom in the graphene lattice, resulting in rehybridization from sp^2^ to sp^3^. Strano demonstrated the successful covalent functionalization of SLG on a Si/SiO_2_ substrate by immersing the sample in an aqueous solution of 4‐nitrobenzenediazonium tetrafluoroborate (4‐NBD), heating it to 40 °C, and allowing the reaction to proceed for 7 hours.^[^ ^13^ ^]^ To exclude the possibility that the observed *D* band enhancement upon laser illumination of the diazonium salt coating follows the same reaction mechanism, a control experiment was performed. A graphene sample was coated with 4‐NBD dissolved in acetonitrile and the solvent was allowed to evaporate, forming a solid compound phase on the graphene surface. The sample was then heated to 40 °C for 5 hours, washed with acetonitrile and isopropyl alcohol, and characterized by Raman spectroscopy (Figure S3).

The absence of a *D* band in the corresponding Raman spectra confirmed that no covalent functionalization occurred through heating alone in this solid‐phase reaction environment. This finding highlights the necessity of laser irradiation for the functionalization in our solid‐phase/solid‐phase protocol. Furthermore, it underlines the inertness of the diazonium salt coating towards thermal activation. Subsequently, Strano's solution‐based reaction conditions^[^ ^1^ ^]^ were applied to 4‐TBBD (see SI: *Functionalization of Single Layer Graphene (SLG) on Si/SiO_2_ in Solution*). As demonstrated in the Raman spectrum in Figure 1c, treating SLG in a heated aqueous solution of 4‐TBBD resulted in covalent functionalization of the graphene lattice, corroborating Strano's observations. However, when adapting these conditions to our solid‐phase/solid‐phase reaction conditions, where SLG was coated with 4‐TBBD and heated to 50 °C for 30 minutes, no functionalization was observed, as confirmed by the absence of a *D* band in Figure 1d. The complete set of reference experiments on the thermal treatment of a 4‐TBBD coated graphene sample is provided in the Supporting Information (see: *Temperature Treatment of a 4‐TBBD Coating on SLG*).

It is important to highlight, though, that heating the diazonium salt coating to 40 °C for 3 hours still allowed for successful laser‐triggered covalent functionalization of graphene (Figure S5), proving that the coating retains its activity even after prolonged heating at moderate temperatures. Nevertheless, upon heating the sample to higher temperatures (100 °C), clear evidence of coating degradation is observed, as further demonstrated in Figure S4b. The stability of the coating is further demonstrated by the absence of any reaction upon reductive activation with Na/K. Traditionally, SLG is activated with Na/K prior to the addition of the functionalization reagent.^[^ ^24^ ^]^ However, when graphene was pre‐coated with a diazonium film, reductive activation using a Na/K alloy to initiate functionalization did not result in a detectable *D* band (Figure S6), indicating no covalent attachment of aryl moieties. These results clearly highlight a significant difference between our solid‐phase system and conventional aqueous functionalization routes. Functionalization in our system requires an external activation of the surface‐bound diazonium salt via laser irradiation; neither thermal treatment nor reductive activation alone leads to any reaction. Furthermore, it is worth mentioning that our laser‐based approach yields comparable or even higher levels of functionalization (*I_D_/I_G_* 2.62, *I_2D_/I_G_* 0.78) to Strano's classic solvent‐based approach (*I_D_/I_G_* 2.28, *I_2D_/I_G_* 0.80).

**Figure 2:**
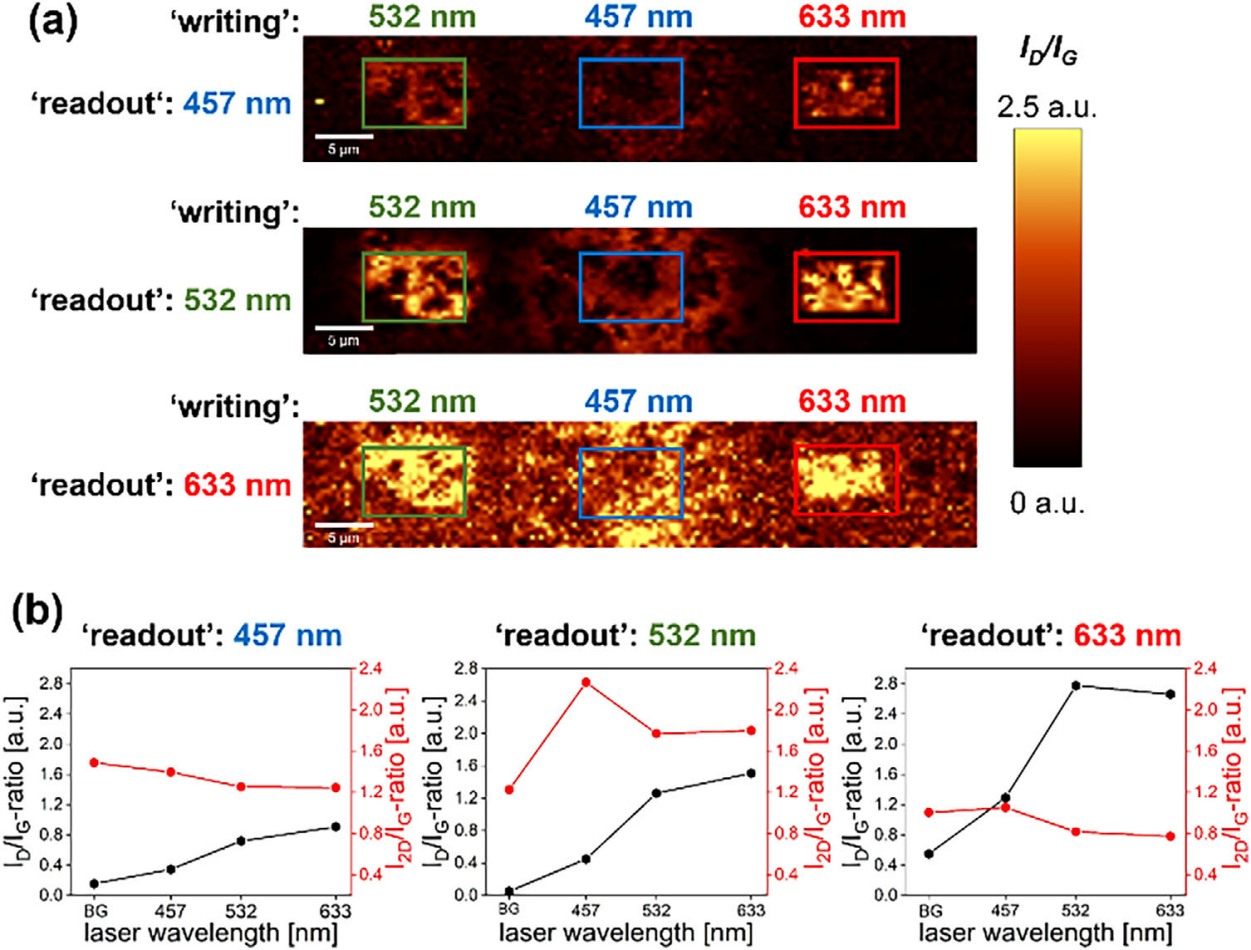
a) Laser excitation wavelength study (4‐TBBD) using three different wavelengths for laser‐triggered covalent functionalization “writing” and spectral “readout” measurements for 457 nm, 532 nm, and 633 nm. For detailed experimental information, see SI: *Laser Excitation Wavelength Study*. b) Evolution of the weighted *I_D_*/*I_G_* and *I_2D_*/*I_G_* ratios for background (BG) and different laser “writing” excitation wavelengths.

As described by Gerein et al.^[^ ^23^ ^]^ and Nagel et al.^[^ ^19^ ^]^ the laser‐triggered covalent functionalization of graphene with suitable precursor molecules (λ^3^ iodanes and dibenzoyl peroxides, respectively) is based on a hot electron injection mechanism of the light‐activated graphene lattice. Since diazonium salts do not exhibit distinct absorption bands in the visible wavelength range (Figure S7), it can be assumed that a similar reaction mechanism is also responsible for their reactivity in laser‐driven reactions. The hypothesis suggests that if the mechanism is valid, there should be no dependence of the amount of covalent addend binding on the chosen laser wavelength. To test this, 4‐TBBD coated samples were irradiated at 10 and 15 mW and 0.05 s using excitation wavelengths of 457 nm, 532 nm, and 633 nm. Here, rectangles were patterned using each respective laser excitation wavelength. Subsequently, the coating was removed by washing, and the laser‐irradiated regions were mapped using each available laser wavelength. The obtained spectral information is presented in Figure 2.

First, it is noticeable that the laser used for the “readout” (and thus its wavelength) has a distinct influence on the spectral information obtained. In the 2D representation of the weighted *I_D_*/*I_G_* ratio, the green laser at 532 nm provides the most clearly defined pattern against the background, which clearly demonstrates the high precision of the covalent bonding of phenyl rings induced by laser‐induced precursor activation. However, it is also evident that the laser wavelength chosen for the laser‐triggered activation of diazonium salts has a significant impact on the achievable degree of functionalization. Assuming that the diazonium salt coating is comparably homogeneous for all three cases considered within the spatially very limited “writing” field, irradiation with 633 nm resulted in an *I_D_*/*I_G_* ratio comparable to that obtained with 532 nm. On the other hand, the use of the blue laser (457 nm) resulted in a drastically reduced activation of the Raman *D* band, and led to a highly scattered introduction of the phenyl moieties. As shown in Figure 2a (central row), a *D* band is measurable, but spatially resolved “writing” becomes impossible. This observation contradicts the aforementioned assumption of a hot electron injection mechanism, which implies a wavelength‐independent activation of the optically active precursor molecules. However, this phenomenon observed in the case of diazonium salts warrants further investigation.

To gain a deeper understanding of other key reaction parameters governing the final degree of covalent functionalization in our “laser writing” approach and to identify the optimal conditions, we systematically varied the applied laser power (*P_L_*) and irradiation time (*t*). In a reference experiment, we initially tested the structural integrity of a substrate‐deposited SLG sheet against laser irradiation with our parameter set (Figure S8). Following the well‐established “laser writing” procedure,^[^ ^19^, ^20^, ^22^, ^23^ ^]^ any unreacted diazonium residues were thoroughly removed by immersing the wafer in THF. This crucial step prevents additional functionalization during the “readout” process. The samples were then analyzed by SRM to determine the respective *I_D_*/*I_G_* ratios. For the parameter optimization, a 532 nm laser was used, with laser powers ranging from 0.1 mW to 20 mW and irradiation times between 0.01 s and 1 s.

**Figure 3:**
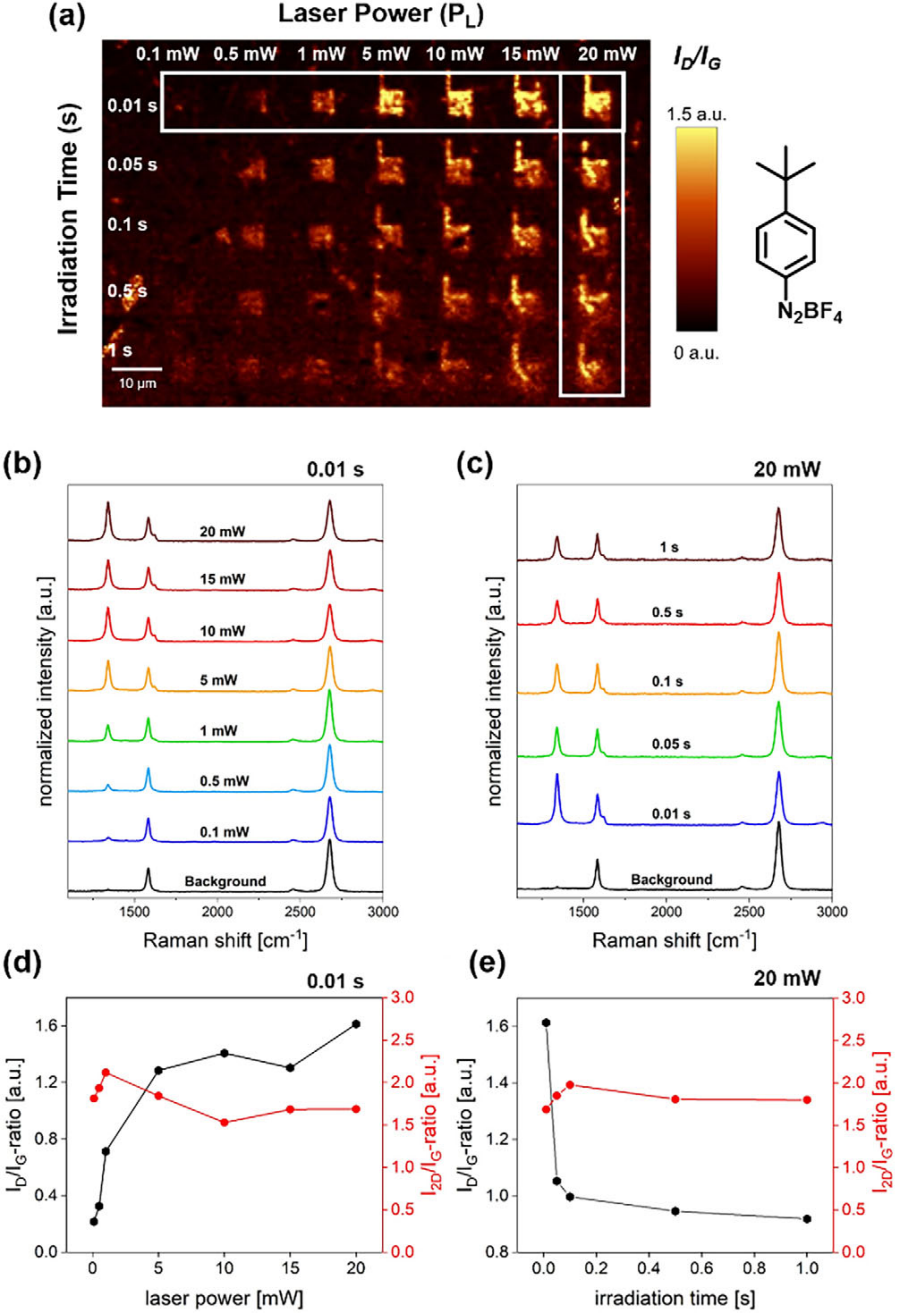
a) Raman “readout” map showing the *I_D_*/*I_G_* ratios of the patterned areas (4‐TBBD) with varying laser power from 0.1 mW to 20 mW and increasing irradiation time from 0.01 s to 1 s. The white rectangles indicates the area from which the Raman spectra in b) and c) were extracted (“readout” parameters: λ_exc_ = 532 nm, 4 mW, 0.5 s, 0.5 µm step size). Evolution of the weighted and normalized Raman spectra with varying laser powers from 0.1 mW to 20 mW for constant irradiation time of b) 0.01 s and varying irradiation times from 0.01 s to 1 s for constant laser power of c) 20 mW, and the evolution of the *I_D_*/*I_G _* ratios and *I_2D_*/*I_G_* ratios with varying laser power for constant irradiation time of d) 0.01 s and varying irradiation times from 0.01 s to 1 s for constant laser power of e) 20 mW.

Figure 3a shows the corresponding Raman “readout” maps with the respective *I_D_*/*I_G_* ratios obtained from the large‐scale Raman sample mapping. The map highlights the regions irradiated by different laser powers and irradiation times, appearing as bright areas against a dark, nonirradiated background. This clear distinction between functionalized areas and the background demonstrates the precision of the laser‐activated functionalization approach, which enables spatially resolved patterning at the micrometer scale. The normalized mean Raman spectra of the functionalized areas with an exposure time of 0.01 s and with increasing laser powers is shown in Figure 3b. The evolution of the Raman spectral information at a laser power of 20 mW with increasing irradiation time is depicted in Figure 3c. Figure 3d,e show the evolution of the respective *I_D_*/*I_G_* and *I_2D_*/*I_G_* ratios. The full set of data is presented in Figure S10 and SI1.

**Figure 4:**
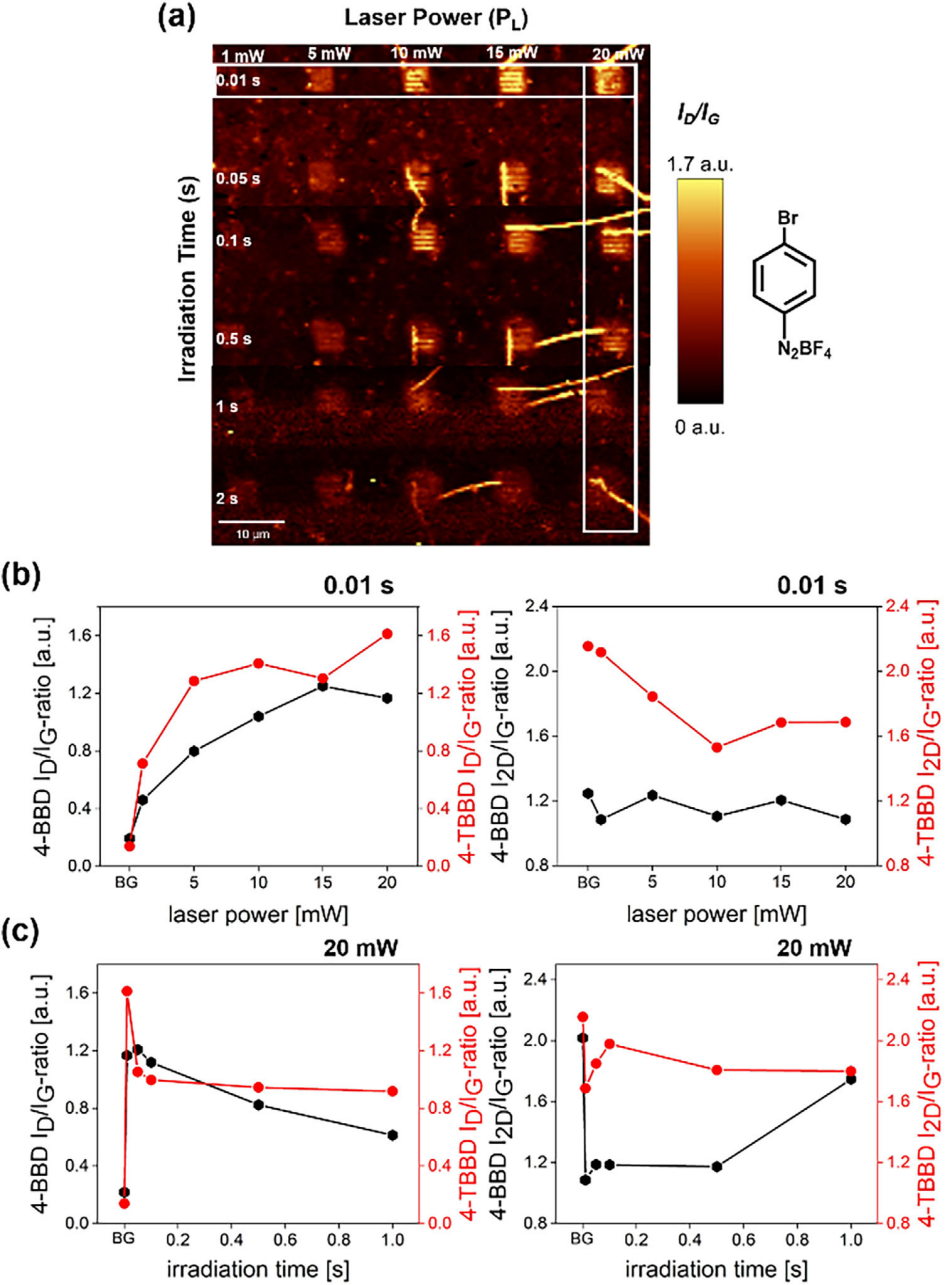
a) Raman “readout” map showing the *I_D_*/*I_G_* ratios of the patterned areas (4‐BBD) with varying laser power from 1 mW to 20 mW and increasing irradiation time from 0.01 s to 2 s. The white rectangles indicates the area from which the Raman spectra in b) and c) were extracted (“readout” parameters: λ_exc_ = 532 nm, 4 mW, 0.5 s, 0.5 µm step size). b) Evolution of the *I_D_*/*I_G_* ratios and *I_2D_*/*I_G_* ratios for 4‐TBBD and 4‐BBD with varying laser power for constant irradiation time of 0.01 s. c) The evolution of the *I_D_*/*I_G_* ratios and *I_2D_*/*I_G_* ratios for 4‐BBD and 4‐TBBD with irradiation times from 0.01 s to 1 s for constant laser power of 20 mW.

For an irradiation time of 0.01 s, increasing the laser power leads to a simultaneous increase in the intensity of the defect‐induced *D* band, with high *I_D_*/*I_G_* ratios of about 1.6 being achieved at 20 mW (Figure 3b,d). In addition, the *D′* band becomes observable above 5 mW, a characteristic feature for covalent addend binding to a relatively high extent. The *2D* band intensity roughly remains unaffected when the laser power is increased. This observation leads to the conclusion that the degree of covalent functionalization obtained by the laser‐triggered diazonium salt activation approach can be continuously enhanced at a given laser irradiation time when the respective laser power is increased. Additionally, an extension of the irradiation time at low laser powers (0.1 mW) also results in an increase in the *I_D_*/*I_G_* ratio. This clearly contrasts with the observations when irradiation time is increased at high laser power (Figure 3c,e). Here, the respective *D* band intensity decreases with increasing irradiation time. Simultaneously, the corresponding *2D* band intensity remains constant. A reduction of the *D* band intensity with no alteration of 2D band intensity and their respective full width at half maximum (FWHM) can be interpreted as a rehybridization of graphene lattice carbon atoms from sp^3^ in sp^2^ configuration. In essence, this is a first indication that the covalently bound phenyl groups can be defunctionalized by extended laser irradiation at higher laser powers, opening the door for laser‐path guided patterned functional group “erasure”. This rationale is corroborated by the Raman spectroscopic data interpretation of the 4‐BBD sample (Figure 4), which yields consistent conclusions (for full set of data, see Figure S13‐S14) – further experimental data supporting this hypothesis are discussed below. It has to be mentioned that the laser‐triggered functionalization of a 4‐MBD film on graphene leads to a different picture. Here, a partial degradation of the graphene lattice can be observed (Figure S15‐S16). This observation will be discussed in more detail below.

**Figure 5:**
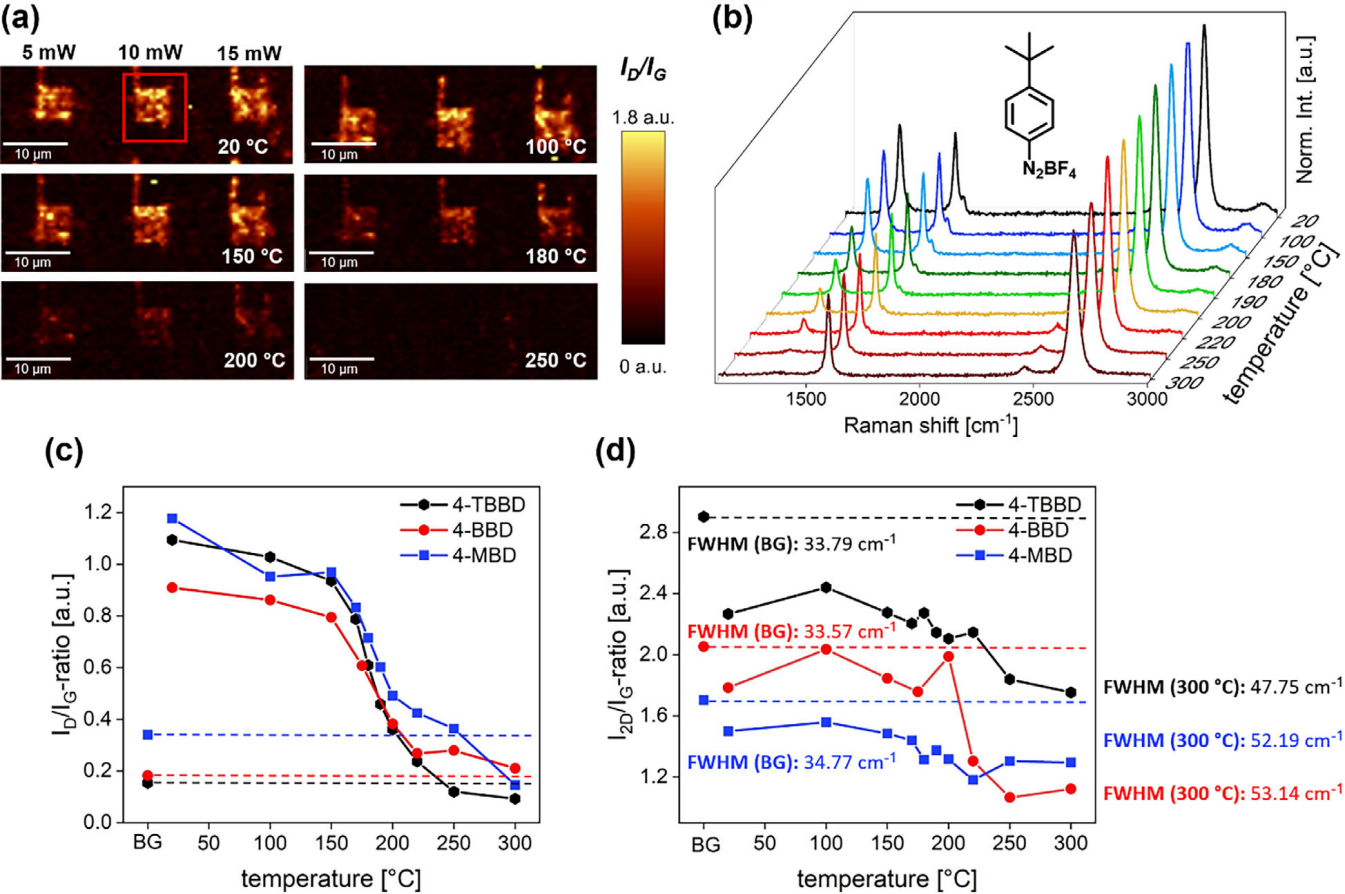
Temperature‐dependent defunctionalization study. a) *I_D_*/*I_G_* ratio maps of an area functionalized with 4‐TBBD at different temperature ranging from 20 °C to 250 °C (“writing” parameters: λ_exc _= 532 nm, 5 mW ‐ 15 mW, 0.01 s, 1 accumulation, 0.5 µm step size, “readout” parameters: λ_exc _= 532 nm, 10 mW, 0.5 s, 1 accumulation, 0.5 µm step size). The red rectangle indicates the extracted area for data presentation in b). b) Weighted and normalized Raman spectra at different temperatures between 20 °C and 300 °C. c) Evolution of the *I_D_*/*I_G_* ratio for the background (BG) and with increasing temperature for 4‐TBBD, 4‐BBD, and 4‐MBD. d) Evolution of the *I_2D_*/*I_G_* ratio for the background (BG) and with increasing temperature for 4‐TBBD, 4‐BBD, and 4‐MBD and FWHM for BG and 300 °C, respectively, for all compounds.

To investigate the thermal stability of covalently patterned graphene areas and gain insights into the reversibility of the covalent phenyl ring binding, we conducted a temperature‐dependent Raman investigation. A 4‐TBBD laser‐patterned sample was gradually heated in a Linkam stage from room temperature to 300 °C, while simultaneously monitoring the Raman spectral information of three covalently functionalized graphene areas created by laser irradiation at 5 mW, 10 mW, and 15 mW, respectively (Figure 5a). The corresponding data analysis for the other two diazonium salts is presented in Figures S17 and S18.

In the temperature regime up to 150 °C, the Raman area mapping does not show any significant alteration in the respective *I_D_*/*I_G_* ratios (Figure 5b,c). Starting at around 150 °C, a progressive decrease in the intensity of the *D* band can clearly be detected, indicative of a detachment of the bound aryl moieties and a rehybridization of the lattice carbon atoms from sp^3^ to sp^2^. This observation aligns with the *D* band intensity development of 4‐BBD and 4‐MBD (data integrated in plot Figure 5c), leading to the conclusion that the chemical nature of the substituent in the *para* position has no distinct influence on the carbon‐carbon bond cleavage between the graphene lattice and the attached phenyl ring. The temperature‐induced defunctionalization process is complete when 300 °C is reached. However, it must be noted that the development of the FWHM of the *2D* band (Figure 5d) indicates that thermal annealing does not lead to complete healing of the introduced sp^3^ defect sites, as the initial value (FWHM (4‐TBBD)  = 33.79 cm^−1^) is not reached even at 300 °C.

**Figure 6:**
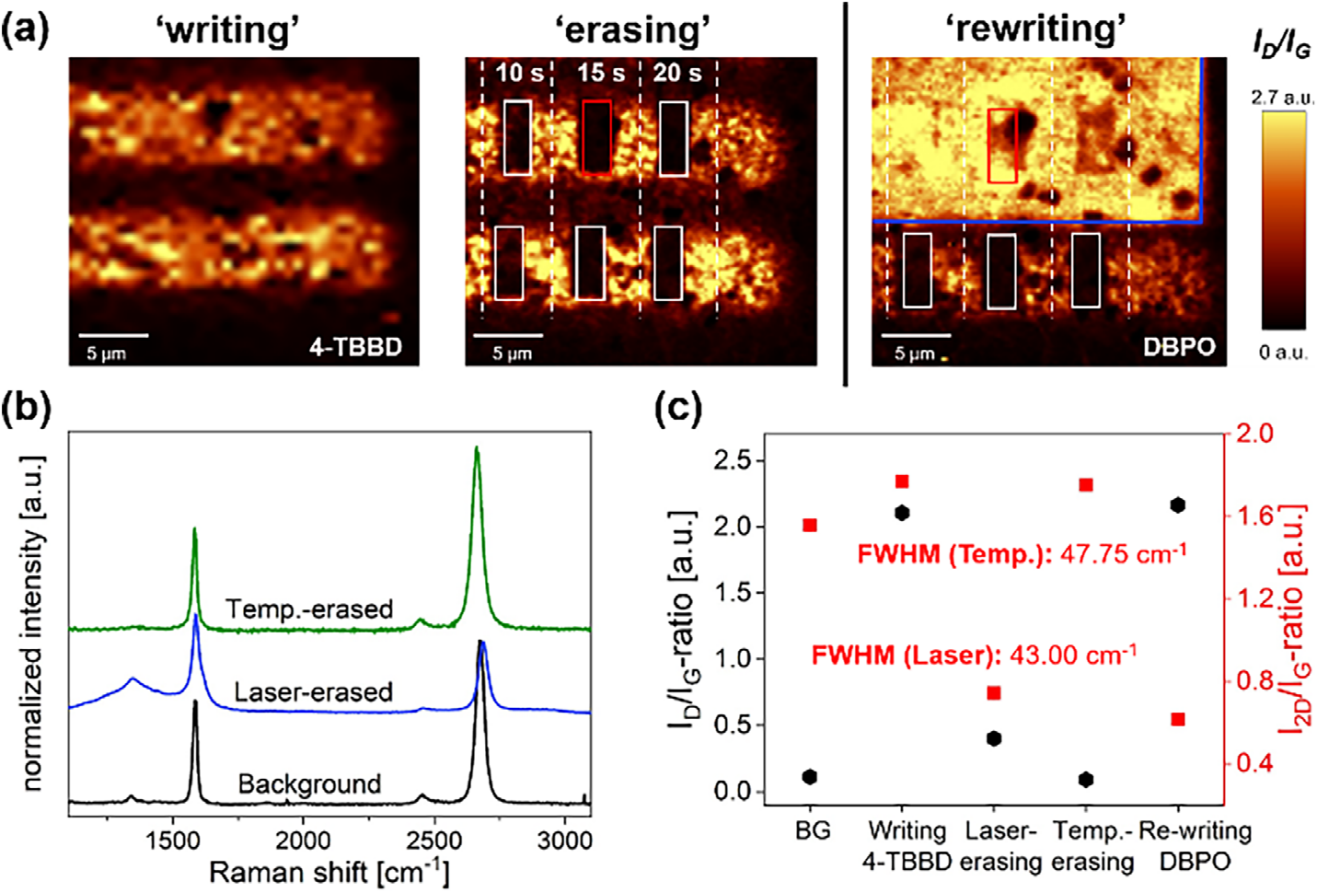
a) Laser‐induced “writing” and defunctionalization using high laser power and prolonged irradiation time (**left**), and “rewriting” in the “erased” areas using DBPO (**right**) ‐ parameters are detailed in the SI. The white rectangles mark the laser‐“erased” areas; red rectangles mark the extracted areas for the spectral information in **b),** blue rectangle marks the “rewriting” with DBPO. b) Weighted and normalized Raman spectra for the unmodified background, for laser‐“erased” area (red rectangle), and temperature‐induced defunctionalization (300 °C). c) Evolution of the *I_D_*/*I_G_* and *I_2D_*/*I_G_* ratios obtained for unmodified background (BG), “writing” with 4‐TBBD, “laser‐erasing”, temperature‐induced defunctionalization (300 °C), and “rewriting” with DBPO, along with the respective FWHM for the laser‐based defunctionalization in comparison to temperature‐based defunctionalization.

As outlined above, higher laser powers (*P_L_*) with prolonged irradiation times lead to a decrease in the *D* band intensity (see Figures 3 and 4), suggesting that covalently bound phenyl groups can be detached by extended laser irradiation. To investigate this intriguing possibility of a laser‐guided, high‐precision functional group removal and to compare the respective spectral information to the classical temperature‐induced defunctionalization scenario, we used a 4‐TBBD functionalized sample and selectively irradiated specific regions of it. The laser‐based “erasing” was performed in area scan mode using a laser power of 20 mW, varying the irradiation time from 10 s to 20 s, with 1 accumulation. As clearly shown by the Raman I_D_/I_G_ areal maps depicted in Figure 6a (left), the D band intensity can be selectively reduced close to zero with high local precision in preselected specific areas by prolonged laser irradiation at 20 mW.

The weighted and normalized Raman spectrum for the laser‐“erased” area (red rectangle) is presented in Figure 6b. The direct comparison of the spectral data obtained by laser‐triggered defunctionalization reveals that, with respect to the behavior of the *2D* band intensity and the FWHM of the *2D* band (Figure 6c), this novel approach is highly competitive with the classic temperature‐induced scenario. Moreover, this exciting finding allows for patterned functional group removal, where the restoration of the intact graphene lattice can be selectively achieved in arbitrarily chosen, laser‐irradiated patterns. This presents a clear advantage over classical thermal graphene annealing (discussed above), as the latter always leads to an uniform functional group removal across the entire framework without specific regional targeting. Furthermore, the question arises whether the laser‐“erased” areas can also be used for targeted functional group “rewriting” as already shown by our group for DBPO.^[^ ^25^, ^26^ ^]^ Therefore, we used our 4‐TBBD sample (Figure 6a), which exhibited the laser‐defunctionalized rectangular regions, and coated it with a DBPO film (for detailed experimental conditions, see SI: *Laser‐Initiated “Erasing” and “Rewriting”*). Afterwards, the laser‐“erased” sample area was relocated, and the upper part of the sample (initially unfunctionalized background, 4‐TBBD‐functionalized area, and laser‐defunctionalized areas) was irradiated by a green laser (λ_exc_  =  532 nm, 0.5 mW, 1 s irradiation. 1 accumulation, and 0.5 µm step size).^[^ ^19^ ^]^ This led to homogeneous *D* band activation (Figure 6a **(right)**) in all laser‐illuminated areas, proving the rationale that a laser‐“erased” graphene area can be successfully re‐addressed in a second laser‐initiated covalent functionalization approach. This opens the possibility for creating complex and mixed‐functionalized graphene architectures by laser‐guided, selective “writing”, “erasing”, and “rewriting” of arbitrary functional moiety bound patterns.

**Figure 7:**
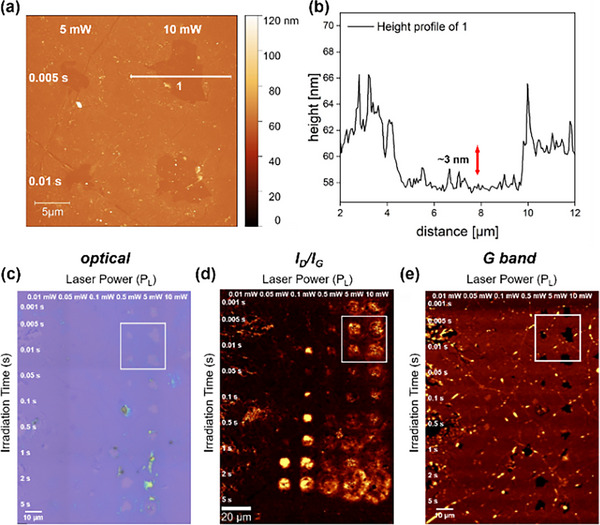
a) AFM image of a section of graphene functionalized with 4‐MBD (*P_L_* > 5 mW) and b) the corresponding height profile showing the degradated graphene lattice with a drop in the height profile of ∼3 nm. c) Corresponding optical image. Raman “readout” maps showing the d) *I_D_*/*I_G_* ratios and e) the *G* band intensity of the patterned area (4‐MBD) with varying laser power from 0.01 mW to 10 mW and increasing irradiation time from 0.001 s to 5 s. The white rectangles indicate the area in which the AFM image in a) was recorded (“readout” parameters: λ_exc_ = 532 nm, 4 mW, 0.5 s, 0.5 µm step size).

To gain deeper insights into the topographical and electronic changes of the functionalized graphene lattice, we conducted atomic force (AFM) and Kelvin‐probe force microscopy (KPFM) measurements (Figure S19‐S21). The AFM image in the 4‐TBBD functionalized graphene sample revealed no significant height alterations between the covalently patterned areas and the unmodified background, a result expected given that the height difference is limited to the thickness of the introduced monomolecular phenyl ring layer. KPFM, however, is anticipated to provide more information, as this characterization tool probes the potential differences between the covalently bound entities and the extended sp^2^ carbon background of graphene. Specifically for the bromo‐ and methoxy‐substituted derivatives, the patterned regions, clearly detectable by Raman areal mapping, should also be visualized by a potential scan with KPFM. This is expected due to a *para*‐substituent‐induced localized decrease and increase in electron density, respectively, in contrast to the “neutral” background. As the data in Figure S20 and S21 show, we were unfortunately not able to resolve the small potential differences in the corresponding areas for either 4‐BBD or 4‐MBD with our system. Surprisingly, however, upon closer AFM examination of the sample functionalized with 4‐MBD, we found that holes had appeared in the otherwise intact graphene layer (Figure 7a,b), precisely in the areas that we had previously irradiated with high laser powers (*P_L_* > 5 mW) in the course of our laser‐based covalent functionalization parameter study.

These holes are also visibly detectable in the corresponding optical image depicted in Figure 7c. The respective Raman *I_D_*/*I_G_* “readout” map (Figure 7d) of the 4‐MBD sample also shows clear differences in contrast to the corresponding data presentations for 4‐TBBD and 4‐BBD (see Figures 3 and 4). Here, for high laser powers and long irradiation times, no clearly defined structural demarcation between the unmodified background and the areas with high *D* band intensity can be detected. Furthermore, a plot of the *G* band (Figure 7e) clearly shows the complete absence of any graphitic/graphene material in these specific areas, in full agreement with the AFM data. We also noticed that the applied 4‐MBD film cannot be removed as easily as in the cases of 4‐TBBD and 4‐BBD by THF/IPA washing. In the regions irradiated with high laser power/long irradiation time, polymeric material, clearly visible, remained on the graphene. This material could only be removed by ultrasonic treatment in acetone, which then led to a partial destruction of the graphene layer in the polymer‐covered areas. In a control experiment (see SI: *Laser “Writing” on Pristine SLG using High Laser Powe*r), we confirmed that irradiation of pristine SLG with high laser powers (*P_L_* > 5 mW) – even with additional sonication – does not disturb the integrity of the graphene lattice (Figure S22). In our opinion, this laser‐initiated polymer formation can be attributed to two main factors: (a) 4‐MBD does not lead to thin, homogeneous films when spin‐coated onto SLG; therefore, more reactive material is applied on top of the sp^2^ graphene lattice. (b) The electron‐donating substituent in 4‐MBD increases the reactivity of the intermediately generated aryl radical, which favors a competitive polymerization reaction. In any case, it should be noted that both the morphology of the applied film and the electronic structure of the applied diazonium compound can have a significant influence on the degree of functionalization achieved and the morphology of the patterned functionalized graphene architecture.

## 3 Conclusion

We have introduced an efficient laser‐guided protocol for the covalent functionalization, defunctionalization, and refunctionalization of SLG using a series of aryl diazonium salts with varying electronic properties. Our method allows for a precise, spatially resolved, laser pathway‐controlled covalent attachment of aryl moieties to the SLG lattice via the laser‐triggered activation of diazonium salt thin films. The decomposition of diazonium salts under light irradiation yields highly reactive aryl radicals, which then undergo an addition reaction with the underlying graphene lattice. We were able to demonstrate that this covalent functionalization is exclusively laser‐driven in our solid‐phase system, requiring neither thermal activation nor reductive agents, a key distinction from conventional solution‐phase approaches. We demonstrated that the degree of functionalization can be easily controlled by tuning the laser power and irradiation time, achieving functionalization levels comparable to or even surpassing established solvent‐based techniques. Furthermore, we discovered a reversible patterning process, laser‐induced defunctionalization, initiated at higher laser powers and prolonged irradiation times, which allows for additional, high‐precision patterning of the covalently introduced moieties at the micrometer level. The most significant advancement of this work is the demonstration of a complete “write‐erase‐rewrite” cycle. We successfully refunctionalized laser‐“erased” graphene areas through laser‐activation of a DBPO precursor, confirming that the restored graphene lattice can be re‐addressed for subsequent covalent functionalization. This breakthrough enables the creation of highly complex and mixed‐functionalized graphene architectures with unprecedented spatial control. This work not only provides a detailed understanding of laser‐triggered graphene functionalization parameters but also introduces a powerful platform for spatially precise, reversible chemical patterning, opening new avenues for advanced graphene‐based devices.

## Supporting Information

The authors have cited additional references within the Supporting Information. The Supporting Information is available free of charge at https:/xxxxxxxx. Materials and procedures, sample preparation, additional Raman, AFM/ KPFM sample characterization and reference experiments.

## Conflict of Interest

The authors declare no conflict of interest.

## Supporting information



Supporting Information

## Data Availability

The data that support the findings of this study are available at Zenodo at https://10.5281/zenodo.16679340
