# Supplementary material for: Spatially Resolved 2D Laser Writing on Graphene Using Diazonium Salts
Source: Chemistry. 2025 Sep 13;31(58):e02468. doi: 10.1002/chem.202502468 (PMC12531459; doi:10.1002/chem.202502468)
Supplement: Supplementary file 1 — Supporting Information [file CHEM-31-e02468-s001.pdf]

F

Supporting Information

Spatially resolved 2D Laser Writing on Graphene Using Diazonium Salts

Johanna Krüger, Tamara Nagel, Bowen Yang, Frank Hauke, and Andreas Hirsch*

Department of Chemistry and Pharmacy & Center of Advanced Materials and Processes (ZMP), Friedrich-Alexander University of Erlangen-Nürnberg, Nikolaus-Fiebiger-Straße 10, 91058 Erlangen, Germany

E-Mail: andreas.hirsch@fau.de

1. Instrumental Section

1.1 Raman Spectroscopy

All Raman measurements were conducted using a WITec alpha300R confocal microscope, equipped with a motorized xyz stage and a CCD detector (1024 x 27, 80 spectra/s). Unless otherwise stated, a green DPSS laser (λ_{exc} = 532 nm, maximum laser power of 30 mW) was employed, in combination with a 100x Zeiss EC "Epiplan-Neofluar" DIC (NA = 0.90) objective and a 600 grooves/mm diffraction grating. During the optimization process, the laser power was varied between 0.05 mW and 20 mW, and the irradiation time was adjusted between 0.001 s and 20 s. A filter was used to achieve laser powers below 0.1 mW. Data analysis was performed using WITec Project software, and further processing was done with OriginLab 2022.

1.2 Scanning Probe Microscopy (SPM)

SPM was performed using a Bruker Dimension Icon 3 microscope in ScanAsyst tapping mode or KPFM mode. Images were obtained with an AFM SCANASYST-AIR probe (silicon tip on a nitride lever) with a tip radius of 2 nm. The scan size was 30 x 30 µm with a resolution of 1024 x 1024 or 512 x 512 pixels. For KPFM images, Bruker SCM-PIT-V2 probes (platinum-iridium coating) on antimony doped Si cantilever were used, with a scan size of 30 x 30 µm and a resolution of 1024 x 1024 or 512 x 512 pixels. The scan rate was set to 0.1 Hz, 0.2 Hz, or 0.4 Hz, and the tip velocity was set to 10 µm/s. The measurement data were collected using NanoScope software and analyzed with Gwyddion.

1.3 UV-Vis Spectroscopy

For the UV-Vis absorption spectroscopy measurements, solutions of the three diazonium salts were prepared. 4-Bromobenzenediazonium tetrafluoroborate and 4-*tert*-butylbenzenediazonium tetrafluoroborate were dissolved in THF (10^{-4} M), and 4-methoxybenzenediazonium tetrafluoroborate was dissolved in acetonitrile (10^{-4} M). The UV-Vis spectra were measured using a Perkin-Elmer Lambda 1050 UV-Vis-NIR spectrometer using a Hellma Suprasil quartz cuvette with 10 mm path length. Measurements were performed in scan mode from 250 nm to 800 nm, with a data interval of 1 nm/s. The obtained data were analyzed using OriginLab 2022.

2. Experimental Section

2.1 Materials and Chemicals

All chemicals were purchased from Merck Sigma-Aldrich Chemie GmbH, Carl Roth GmbH, and VWR International GmbH and were used without further purification. 1 x 1 cm^2 CVD monolayer graphene with a polymethyl methacrylate (PMMA) coating was obtained from ACS Material Inc. (USA) as "trivial transfer graphene". Prior to use, the Si/SiO_2 wafers (300 nm silicon oxide layer, obtained from Frauenhofer e.V.) were cleaned by rinsing with isopropanol (IPA) and acetone.

2.2 Synthesis: 4-*tert*-Butylbenzenediazonium Tetrafluoroborate (4-TBBD)

2 mL (12.60 mmol) 4-*tert*-butylaniline was mixed with 7 mL (111.60 mmol) of HBF_4 (48 wt% in H_2O) in a 250 mL flask. The resulting yellow solid was dissolved in 35 mL of H_2O. The orange solution was cooled in an ice bath while stirring. Then, a solution of $NaNO_2$ (1.70 g, 24.60 mmol) in 3 mL of H_2O was slowly added to the reaction mixture over 30 minutes. The resulting solid was filtered off and washed with cold water. The solid was then recrystallized in 2.5 mL of methanol and cooled to -24 °C overnight. The white crystals were filtered off and washed with cold diethyl ether. The product was left as white crystals. Yield: 1.03 g (4.15 mmol, 33 %).

^{1}H-NMR (400 MHz; CD_3CN; rt): d (ppm) = 1.38 (s, 9H), 7.97 (d, J = 8.0 Hz, 2H), 8.41 (d, J = 8.0 Hz, 2H).

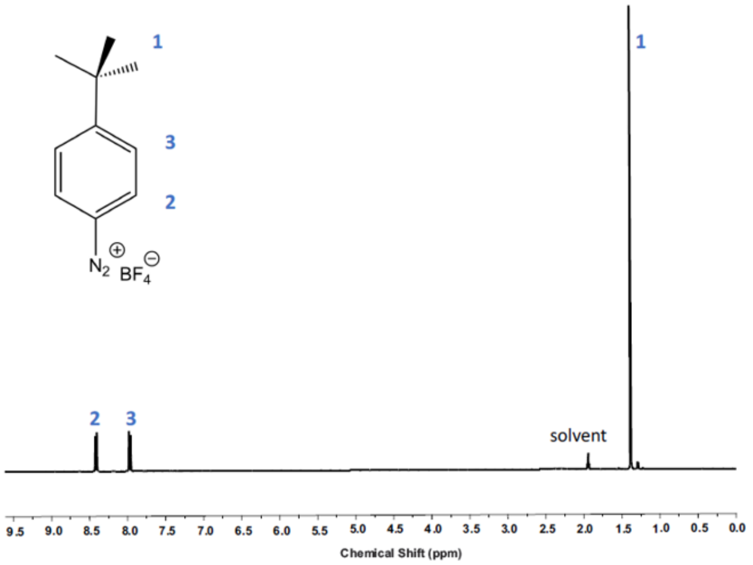

Figure S1: ^{1}H-NMR of 4-TBBD in CD_3CN measured at 400 MHz.

2.3 Single Layer Graphene Sample Preparation – Wet Chemical Transfer

A wet transfer technique was used to transfer CVD graphene covered with a PMMA layer to a Si/SiO_2 wafer (0.5 x 0.5 cm). The graphene layer was immersed in bidistilled water (dd H_2O) to release it from the polymer membrane, where it subsequently floated on the water surface. A pre-cleaned Si/SiO_2 wafer was then used to pull the graphene film from the water, placing it onto the wafer. The sample was subsequently dried by heating at 120 °C for one hour. To remove the PMMA layer, the graphene wafer was exposed to acetone vapor for two hours.

2.4 SLG Graphene Coating Techniques

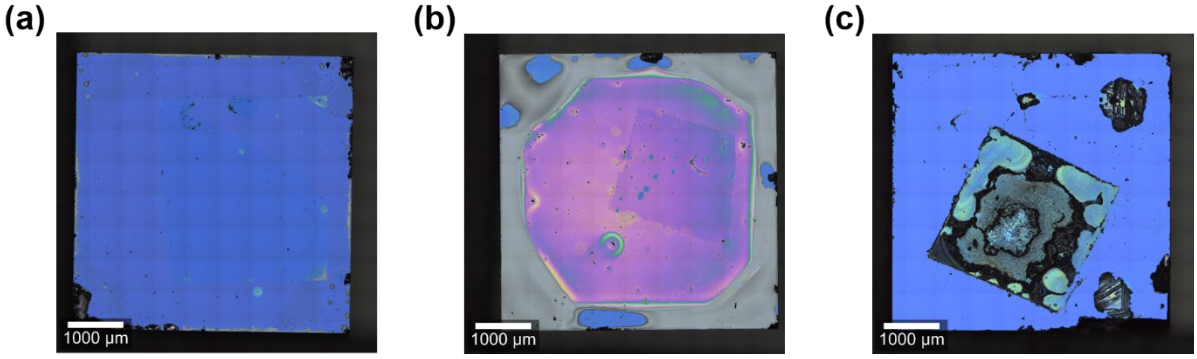

Figure S2: Optical images of graphene coatings on Si/SiO_2 by three different deposition methods: **(a)** 4-TBBD *via* spin-coating, **(b)** 4-BBD *via* PMMA-assisted spin-coating, and **(c)** 4-MBD *via* drop-casting.

2.5 Laser-Triggered Covalent Functionalization of 4-TBBD-Coated Graphene

Raman Single Spectra of SLG on Si/SiO_2: SLG on a Si/SiO_2 wafer was spin-coated with one drop of 4-TBBD in THF ($1\cdot10^{-3}$ M) at 500 rpm for 30 s, resulting in a thin and homogeneous coating. The Raman single-point spectra were measured following the work of Edelthalhammer *et al.*,[1] using a Raman imaging microscope, 0.8 mW laser power, 60 s irradiation time, and 1 accumulation.

2.6 Diazonium Salt Reference Experiments

Temperature Treatment of a 4-NBD Coating on SLG: SLG on a Si/SiO_2 wafer was spin-coated with one drop of 4-NBD in acetonitrile ($1\cdot10^{-3}$ M) at 500 rpm for 2 s. The coated wafer was heated to 40 °C for 5 h. Afterwards, the wafer was washed by

immersing the sample in 4 mL of acetonitrile for 30 minutes, followed by washing with 2 mL of IPA. The sample was characterized by Raman spectroscopy in area scan mode using 5 mW laser power, 0.5 s irradiation time, 1 accumulation, and 0.5 µm step size. The Raman spectra show no *D* band after heating, confirming, that no covalent functionalization occurred (**Fig. S3**).

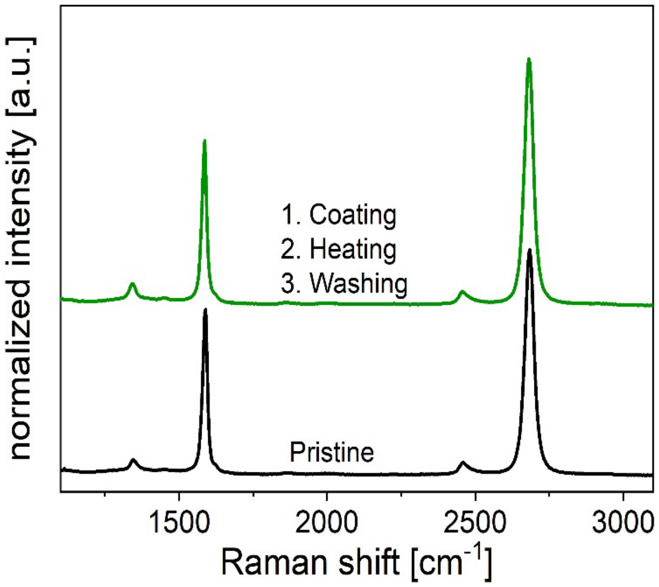

Figure S3: Raman spectra of SLG on Si/SiO_2. **Black:** pristine graphene. **Green**: after coating, heating (40 °C, 5 h), and washing. The spectra were recorded using 5 mW laser power, 0.5 s irradiation time, 1 accumulation, 0.5 µm step size).

Temperature Treatment of a 4-TBBD Coating on SLG: SLG on a Si/SiO_2 wafer was spin-coated with one drop of 4-TBBD in THF ($1\cdot10^{-3}$ M) at 500 rpm for 2 s. The coated wafer were heated for 30 minutes each to 50 °C and 100 °C. Afterwards, the wafer were washed by immersing the sample in 4 mL of THF for 30 minutes, followed by washing with 2 mL of IPA. Raman spectra were taken from **(I)** coated sample before heating, **(II)** coated sample after heating, and **(III)** sample after heating and washing. The Raman spectra of all samples after heating and washing show no *D* band, confirming, that no covalent functionalization occurred by thermal treatment (**Fig. S4**). It is noticeable, that heating the sample to 100 °C for 30 minutes lead to a degradation of the coating. This is observable by no *D* band in the Raman spectra of **(II)** in **Fig. S4b**, whereas heating to 50 °C the coating stays unaffected.

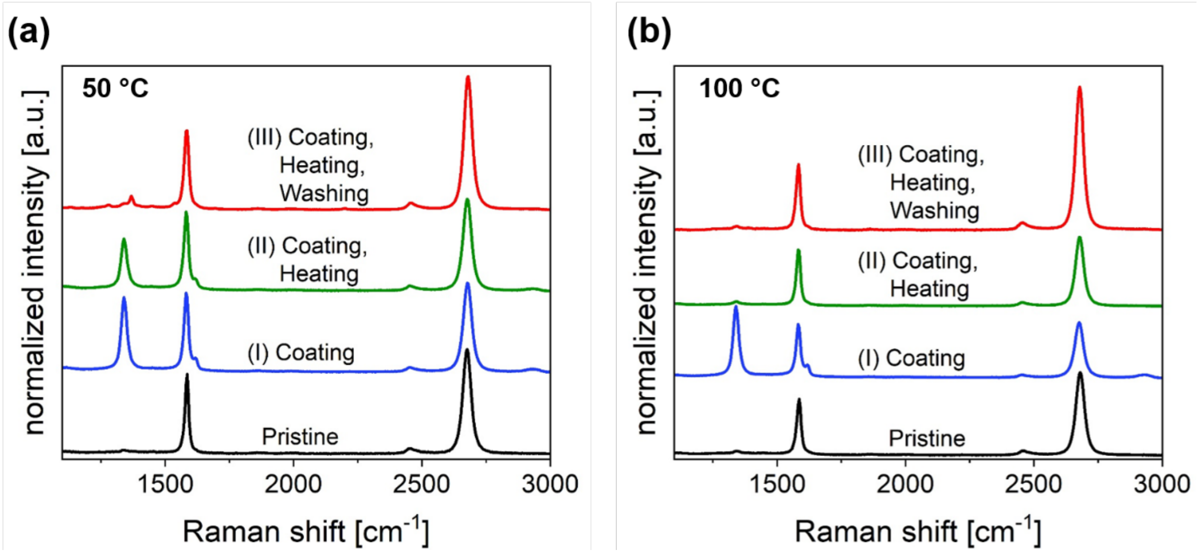

Figure S4: Raman spectra of SLG on Si/SiO_2, coated with 4-TBBD and thermal treatment. **(a) Black:** pristine graphene (single spectrum, 532 nm, 10 mW, 1 s. 1 acc.). **(I)** after coating (averaged spectra, 532 nm, 15 mW, 0.01 s. 1 acc.), **(II)** after heating (averaged spectra, 532 nm, 15 mW, 0.01 s. 1 acc.), **(III)** after heating (50 °C, 30 min) and washing (averaged spectra, 532 nm, 4 mW, 0.5 s. 1 acc.). **(b) Black:** pristine graphene (averaged spectra, 532 nm, 10 mW, 0.05 s. 1 acc.), **(I)** after coating (averaged spectra, 532 nm, 15 mW, 0.01 s. 1 acc.), **(II)** after heating (averaged spectra, 532 nm, 15 mW, 0.01 s. 1 acc.), **(III)** after heating (100 °C, 30 min) and washing (averaged spectra, 532 nm, 4 mW, 0.5 s. 1 acc.).

Functionalization of Single Layer Graphene (SLG) on Si/SiO_2 in Solution: The reaction protocol of Strano *et al.* was adapted for the wafer-scale functionalization of SLG on a Si/SiO_2 wafer.[2] 4-*tert*-Butylbenzenediazonium tetrafluoroborate (21 mg, $1.7{\cdot}10^{-2}$ M) and an aqueous solution of 1 wt% sodium dodecyl sulfate (0.5 mL) were dissolved in 5 mL dd H_2O. SLG on a Si/SiO_2 wafer was immersed in the reactant solution for 17 h at 40 °C with vigorous stirring. Afterward, the wafer was immersed in double distilled H_2O for 4 h and washed with 4 mL of IPA. For characterization, Raman spectroscopy maps were performed in the area scan mode using 5 mW laser power, 1 s irradiation time, 1 accumulation, and 0.5 µm step size.

Temperature Treatment of a 4-TBBD Coating with subsequent Laser-Triggered Covalent Functionalization: SLG on a Si/SiO_2 wafer was spin-coated with one drop of 4-TBBD in THF ($1{\cdot}10^{-3}$ M) at 500 rpm for 2 s. A Raman single-point spectrum was recorded using 15 mW laser power, 1 s irradiation time, and 1 accumulation. The coated wafer was heated to 40 °C for 3 h. Note that there is no washing step between

the sample heating and the subsequent Raman-based characterization. A Raman single-point spectrum was recorded using 15 mW laser power, 1 s irradiation time, and 1 accumulation. The Raman spectra show a prominent *D* band before and after the heating procedure (**Fig. S5**), confirming the coating stability against thermal treatment.

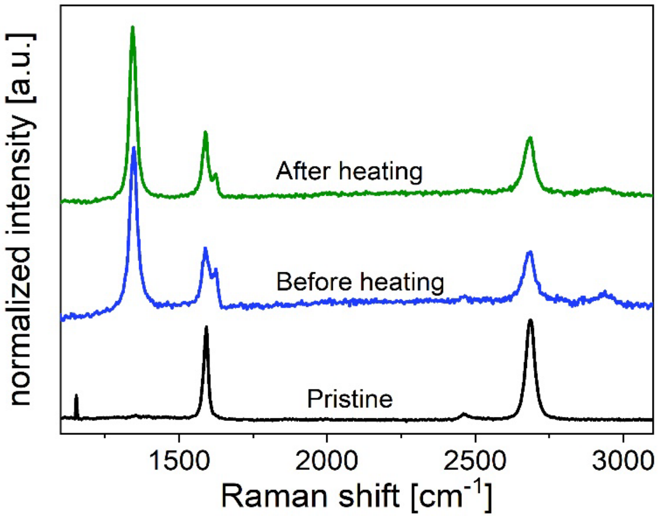

Figure S5: Raman spectra of SLG on Si/SiO_2. **Black:** pristine graphene (5 mW laser power, 0.5 s irradiation time, 1 accumulation, and 0.5 µm step size). **Blue**: with coating of 4-TBBD before heating (15 mW laser power, 1 s irradiation time, and 1 accumulation). **Green**: with coating of 4-TBBD after heating (40 °C, 3 h, 15 mW laser power, 1 s irradiation time, and 1 accumulation).

2.7 Reductive Functionalization of a Coated SLG on Si/SiO_2 using Na/K Alloy

In an argon-filled glove box, 20 mg of sodium and 60 mg of potassium were mixed to obtain the Na/K alloy. Two drops of the Na/K alloy were dissolved in 5 mL of anhydrous 1,2-dimethoxyethan (DME) and stirred for 30 min.

(1.) Na/K, (2.) 4-TBBD: In the classical reductive functionalization route, the SLG on a Si/SiO_2 wafer is immersed in 1-2 mL of the Na/K DME solution for 5 min. The sample is then removed from the solution and placed in a solution with the functionalization reagent 4-TBBD ($9.7 \cdot 10^{-2}$ M in DME) for 5 min. Subsequently, the wafer was washed with DME and the sample was characterized with Raman spectroscopy maps performed in area scan mode using 5 mW laser power, 0.5 s irradiation time, 1 accumulation, and 0.5 µm step size.

(1.) 4-TBBD, (2.) Na/K: A 4-TBBD coated ($1{\cdot}10^{-3}$ M, THF, spin coating, 1 drop, 500 rpm) SLG on a Si/SiO_2 wafer was immersed in the Na/K DME solution for 5 min. Subsequently, the wafer was washed with DME and the sample was characterized with Raman spectroscopy maps performed in area scan mode using 5 mW laser power, 0.5 s irradiation time, 1 accumulation, and 0.5 µm step size.

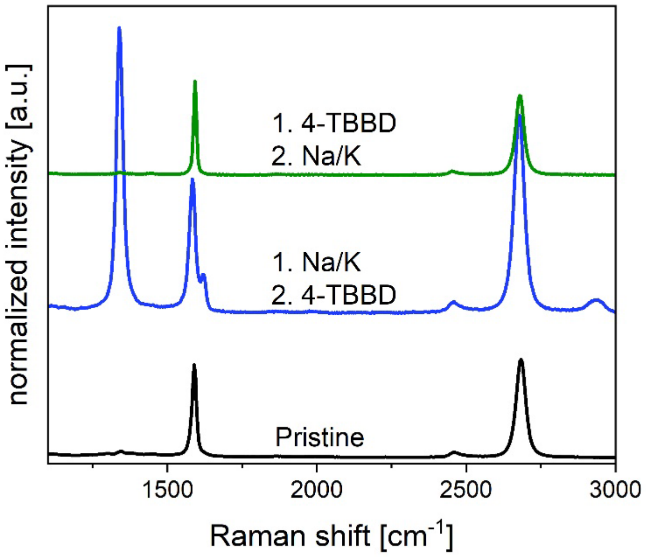

Figure S6: Raman spectra of SLG on Si/SiO_2. **Black:** pristine graphene (5 mW laser power, 0.5 s irradiation time, 1 accumulation, and 0.5 µm step size). **Blue**: classical reductive functionalization: 1. Na/K alloy activation, 2. addition of 4-TBBD (Raman characterization: 5 mW laser power, 0.5 s irradiation time, and 1 accumulation). **Green**: 1. Coating with 4-TBBD, 2. addition of Na/K alloy in DME (Raman characterization: 5 mW laser power, 0.5 s irradiation time, and 1 accumulation).

2.8 UV-Vis Characterization of Applied Diazonium Salts

In the UV-Vis spectra, the diazonium salts used do not show any absorption in the visible region, which excludes a direct activation of the diazonium salts by the employed 532 nm laser. Therefore, a different mechanism is proposed for the reaction performed, in which graphene acts as a photosensitizer, transferring an excited electron to the functionalizing reagent and inducing the decomposition of the diazonium compound. See literature.[3]

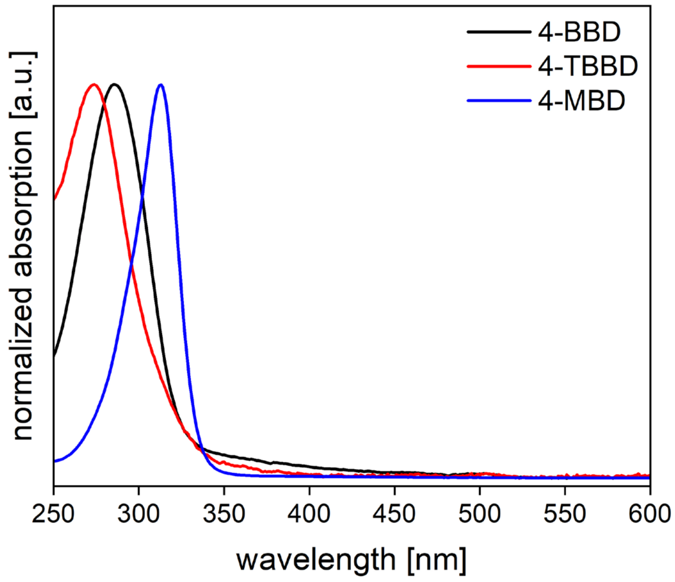

Figure S7: UV-Vis absorption spectra of 4-BBD in THF (10^{-4} M), 4-MBD in acetonitrile (10^{-4} M), and 4-TBBD in THF (10^{-4} M) showing no absorption in the region of the green laser (532 nm).

2.9 Laser Excitation Wavelength Study

For the laser excitation wavelength study, the same Raman instrument was used as described in detail above. In addition to the 532 nm green laser, 457 nm and 633 nm lasers were used to perform the laser 'writing' (**Fig. 2a**, indicated by the colored boxes) and 'readout' measurements using three different laser wavelengths. The laser 'writing' was performed using 4-TBBD ($c = 1 \cdot 10^{-3}$ M in THF, spin-coating, 500 rpm). The 'witings' for 532 nm and 457 nm were performed in area scan mode using 15 mW laser power, 0.05 s irradiation time, 1 accumulation, and 0.5 µm step size. The 'writing' for 633 nm was performed in area scan mode using 10 mW laser power, 0.05 s irradiation time, 1 accumulation, and 0.5 µm step size. After 'writing', the wafer was immersed in 5 mL THF for 30 min and washed with 5 mL THF/IPA. All 'readouts' were performed using the three different laser wavelengths in area scan mode using 5 mW laser power, 0.5 s irradiation time, 1 accumulation, and 0.5 µm step size.

2.10 Laser 'Writing' on Pristine Graphene without Coating

To demonstrate the structural integrity of the graphene sheet under laser irradiation, a pristine graphene sample on a Si/SiO_2 substrate was exposed to the same laser parameters (P_L and t) as those used for functionalization with 4-TBBD. The Raman 'readout' map following irradiation exhibits no increase in the D band intensity within

the exposed region, ruling out defect formation solely due to laser exposure (**Fig. S8a-c**). Additionally, the I_D/I_G, I_{2D}/I_G, and FWHM values for the *2D* band remain consistent across all extracted mean spectra, further confirming the structural integrity of the graphene (**Fig. S8d**).

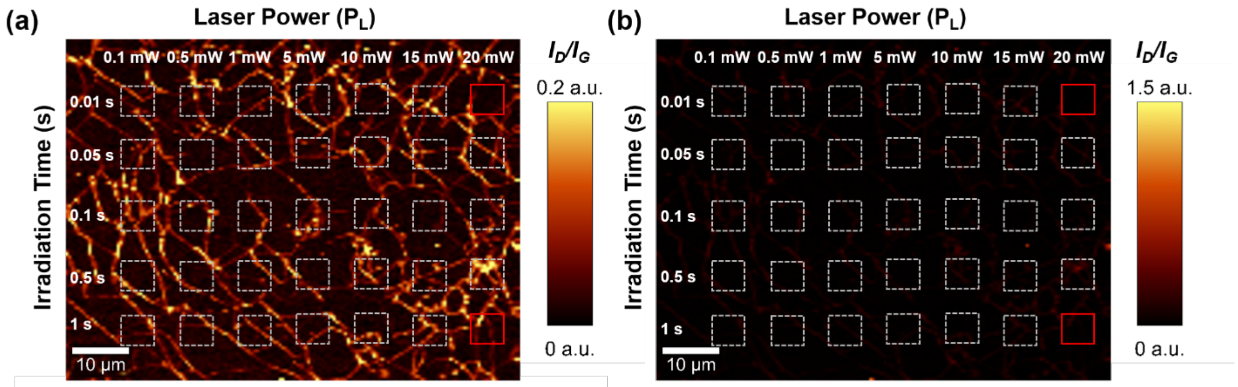

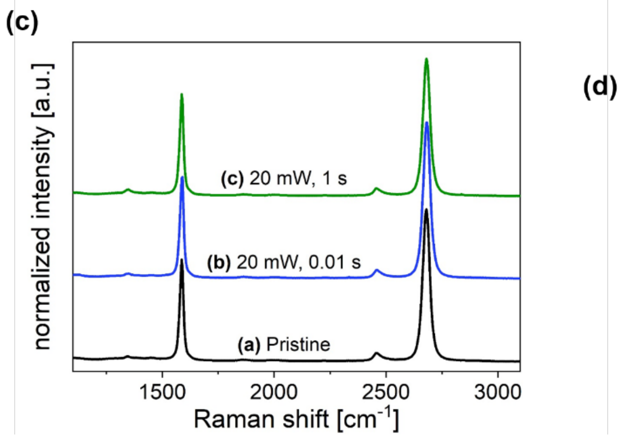

(d)

	I_D/I_G ratio	I_{2D}/I_G ratio	FWHM *2D* band [cm^{-1}]
**(a)**	0.06	1.49	38.78
**(b)**	0.06	1.53	39.21
**(c)**	0.08	1.34	39.03

Figure S8: (a) Raman 'readout' map after laser irradiation using different P_L and *t* exhibiting no *D* band intensity (I_D/I_G ratio from 0 to 0.2 a.u.), showing structural integrity of the graphene sheet. **(b)** Identical Raman 'readout' image with the I_D/I_G ratio scale extended from 0 to 1.5 a.u. (c) Normalized mean Raman spectra extracted from the red marked areas. **(d)** The values for I_D/I_G, I_{2D}/I_G and FWHM remain consistent across all extracted mean spectra.

2.11 Parameter Optimization Study for the Laser-Triggered Functionalization of SLG with Aryl Diazonium Salts

2D Patterning of SLG on Si/SiO₂ with 4-tert-Butylbenzenediazonium Tetrafluoroborate (4-TBBD): SLG on a Si/SiO_2 wafer was spin-coated with 4 drops of 4-TBBD in THF ($2{\cdot}10^{-3}$ M) at 500 rpm for 10 s, resulting in a thin and homogeneous coating (see **Fig. S2a**), which was immediately used for the optimization process to avoid decomposition of the compound. The 'writing' was performed in the area scan mode. The laser power was varied between 0.01 mW and 20 mW and the irradiation time was varied between 0.01 s and 5 s in area mapping with 0.5 µm step size and 10 µm spacing between the squares. After 'writing', the wafer was immersed in 5 mL tetrahydrofuran (THF) for 30 min and washed with 5 mL THF/IPA to avoid background functionalization. The 'readout' measurement was performed in area scan mode using 4 mW laser power, 0.5 s irradiation time, 1 accumulation, and 0.5 µm step size. **Fig. S9** displays the Raman 'readout' map showing the I_D/I_G ratios confirming the binding of *tert*-butyl-substitued phenyl radicals in the irradiated regions. All corresponding Raman spectra are presented in **Fig. S10-S11**.

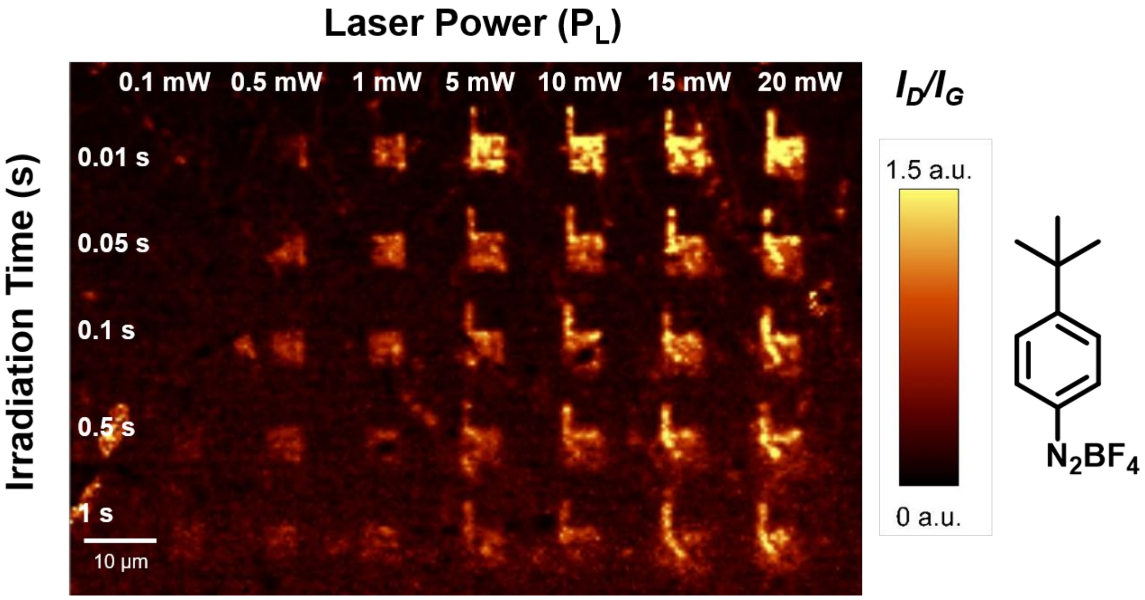

Figure S9: (a) Raman 'readout' map showing the I_D/I_G ratios of the patterned areas with varying laser power from 0.1 mW to 20 mW and increasing irradiation time from 0.01 s to 1 s ('readout' parameters: λ_{exc} = 532 nm, 4 mW laser power, 0.5 s irradiation time, 1 accumulation, and 0.5 µm step size).

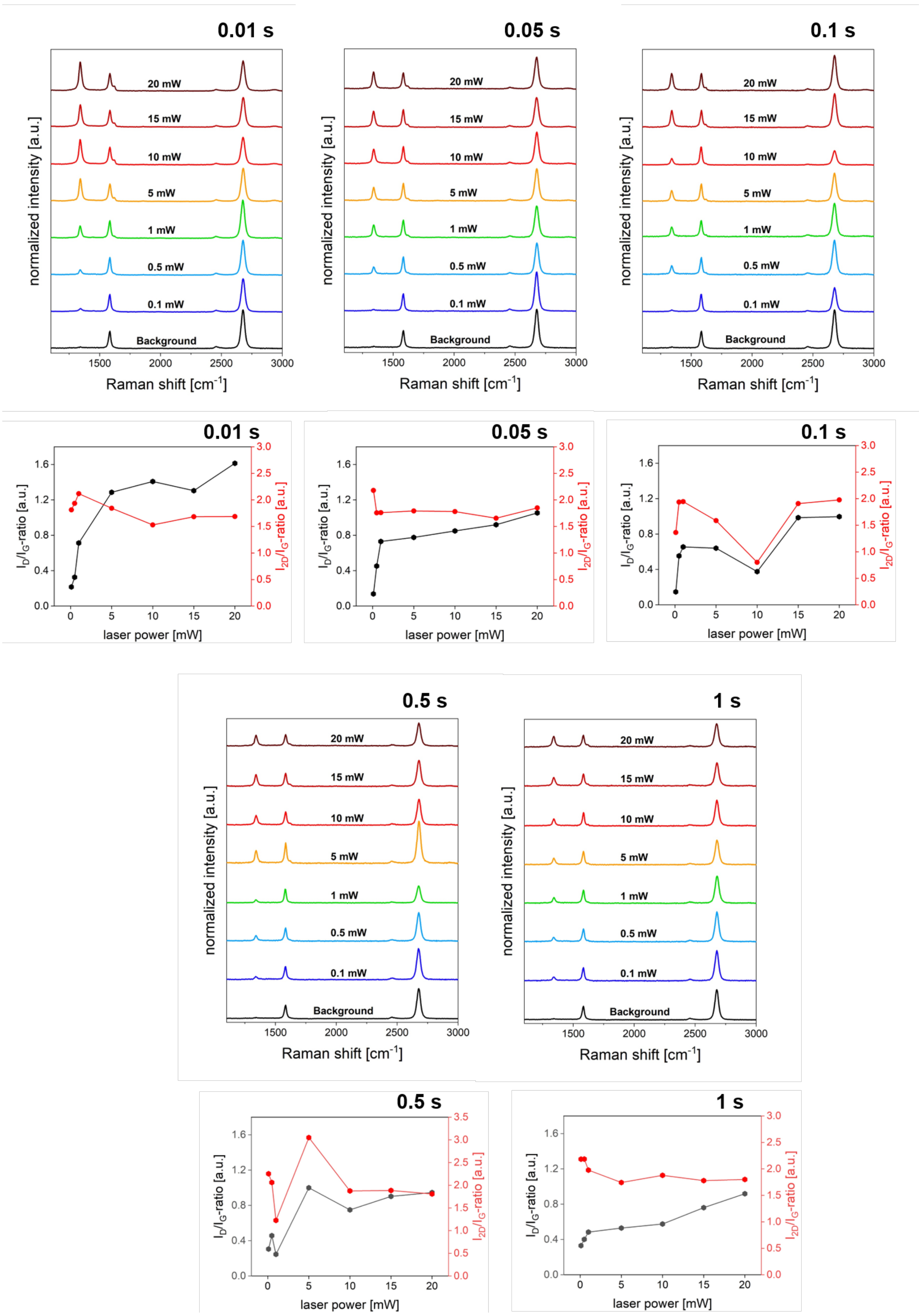

Figure S10: Evolution of the weighted and normalized Raman spectra of laser ‘writing’ with 4-TBBD at varying laser powers from 0.1 mW to 20 mW for constant irradiation times and the corresponding I_D/I_G and I_{2D}/I_G ratios.

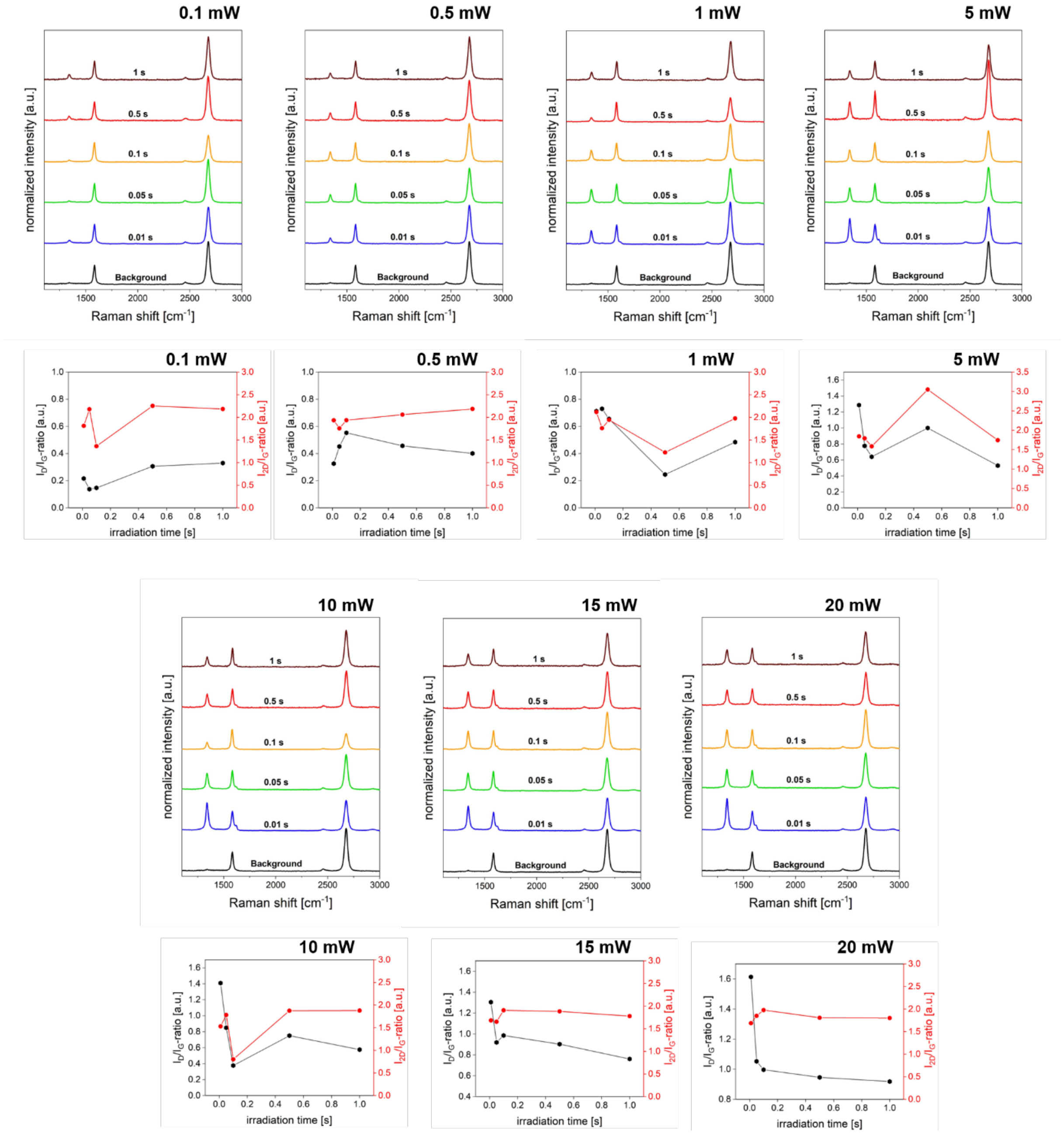

Figure S11: Evolution of the weighted and normalized Raman spectra of laser 'writing' with 4-TBBD at varying irradiation times from 0.01 s to 1 s and a constant laser powers together with corresponding I_D/I_G and I_{2D}/I_G ratios.

2D Patterning of SLG on Si/SiO2 with 4-bromobenzenediazonium tetrafluoroborate (4-BBD) SLG on a Si/SiO_2 wafer was spin-coated with one drop of 4-BBD ($1 \cdot 10^{-3}$ M) and PMMA (0.4 wt%) in THF at 500 rpm for 10 s, yielding a thick, and mostly homogeneous coating (see **Fig. S2b**), which was immediately used for the optimization process to avoid decomposition of the compound. The 'writing' was performed in a stepwise raster operating mode. The laser power was varied between 1 mW and 20 mW and the irradiation time was varied between 0.01 s and 2 s. The patterns were written in a stepwise raster mode with 1 µm step size and 12.5 µm spacing between the squares. After 'writing', the wafer was immersed in 5 mL THF for 30 min and washed with 5 mL THF/IPA to avoid background functionalization. The 'readout' measurement was performed in area scan mode using 4 mW laser power, 0.5 s irradiation time, 1 accumulation, and 0.5 µm step size. **Fig. S12** displays the Raman 'readout' map showing the I_D/I_G ratios confirming the binding of bromo-substituted phenyl radicals in the irradiated regions. All corresponding Raman spectra are presented in **Fig. S13-S14**. Please note that the laser draws additional lines due to certain settings. In future, care will be taken to ensure that the laser is set correctly.

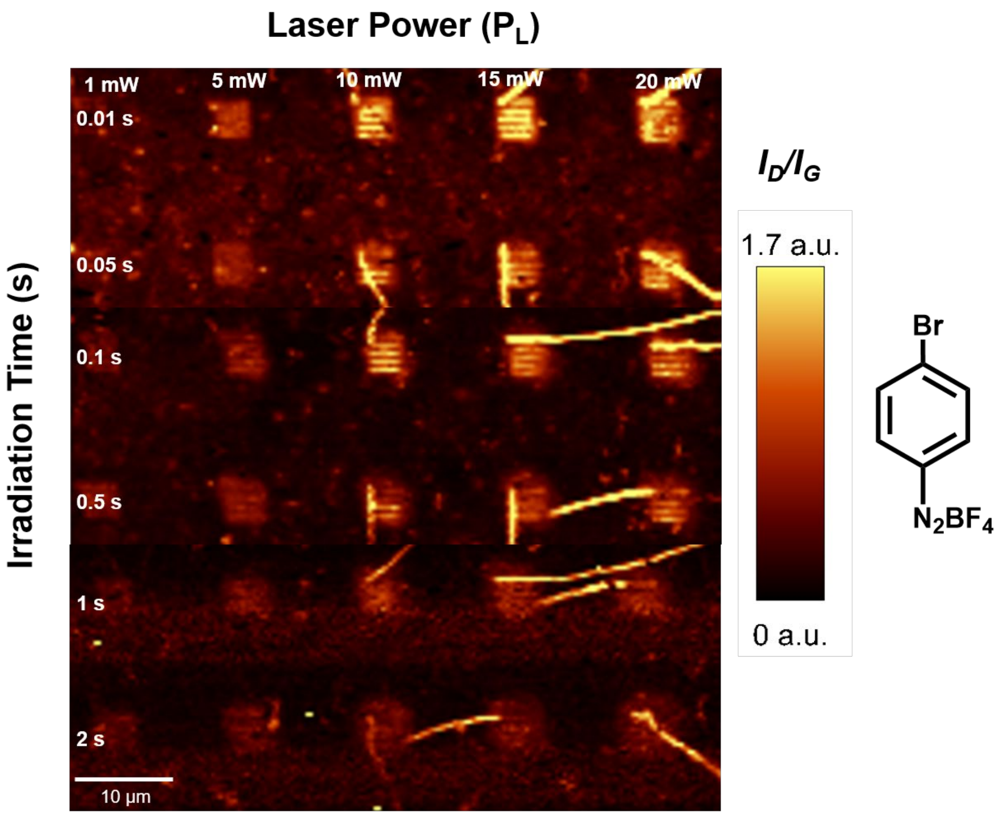

Figure S12: (a) Raman 'readout' map showing the I_D/I_G ratios of the patterned areas with varying laser power from 1 mW to 20 mW and increasing irradiation time from 0.01 s to 2 s ('readout' parameters: λ_{exc} = 532 nm, 4 mW laser power, 0.5 s irradiation time, 1 accumulation, and 0.5 µm step size).

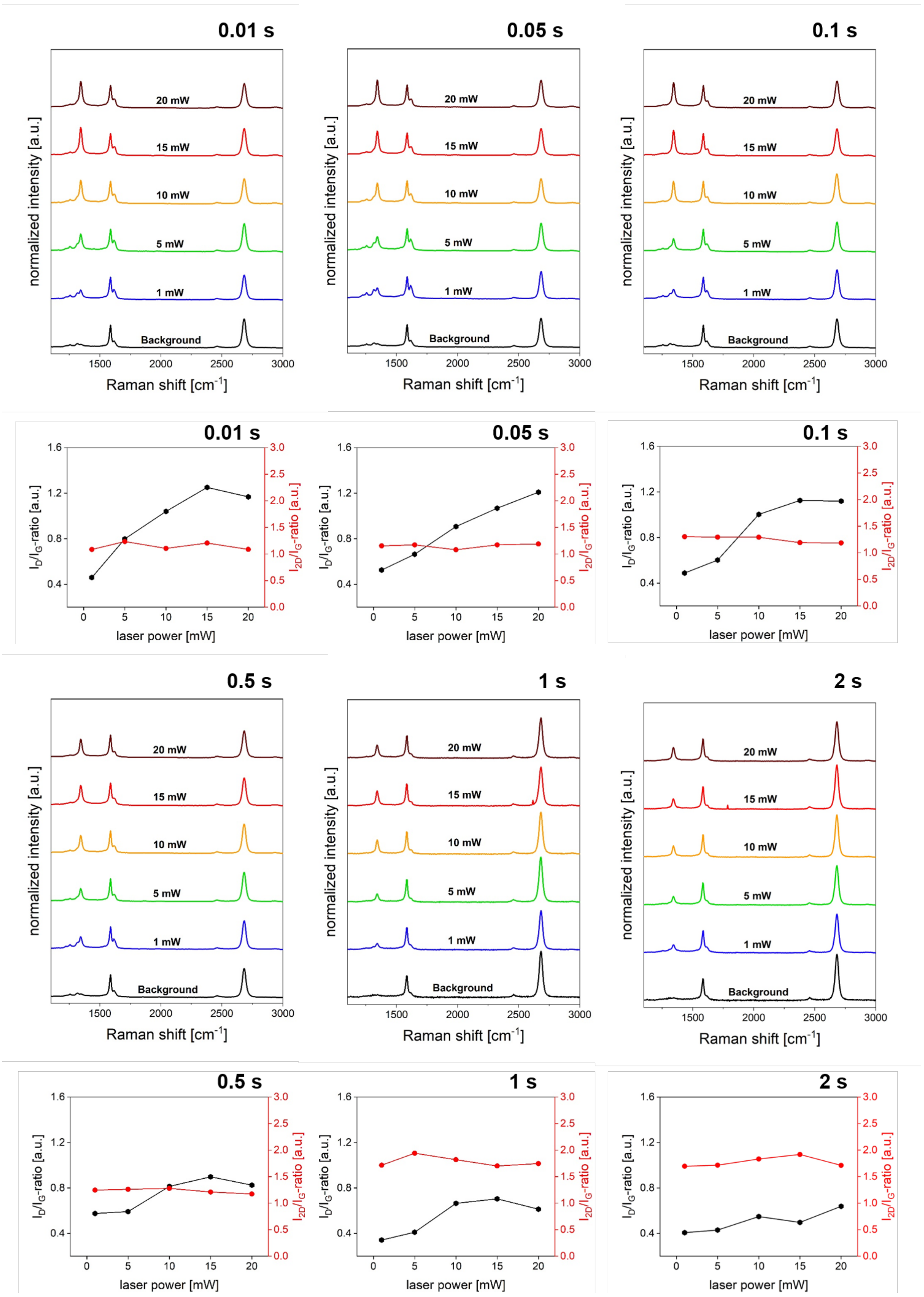

Figure S13: Evolution of the weighted and normalized Raman spectra of laser ‘writing’ with 4-BBD at varying laser powers from 1 mW to 20 mW for constant irradiation times and corresponding I_D/I_G and I_{2D}/I_G ratios.

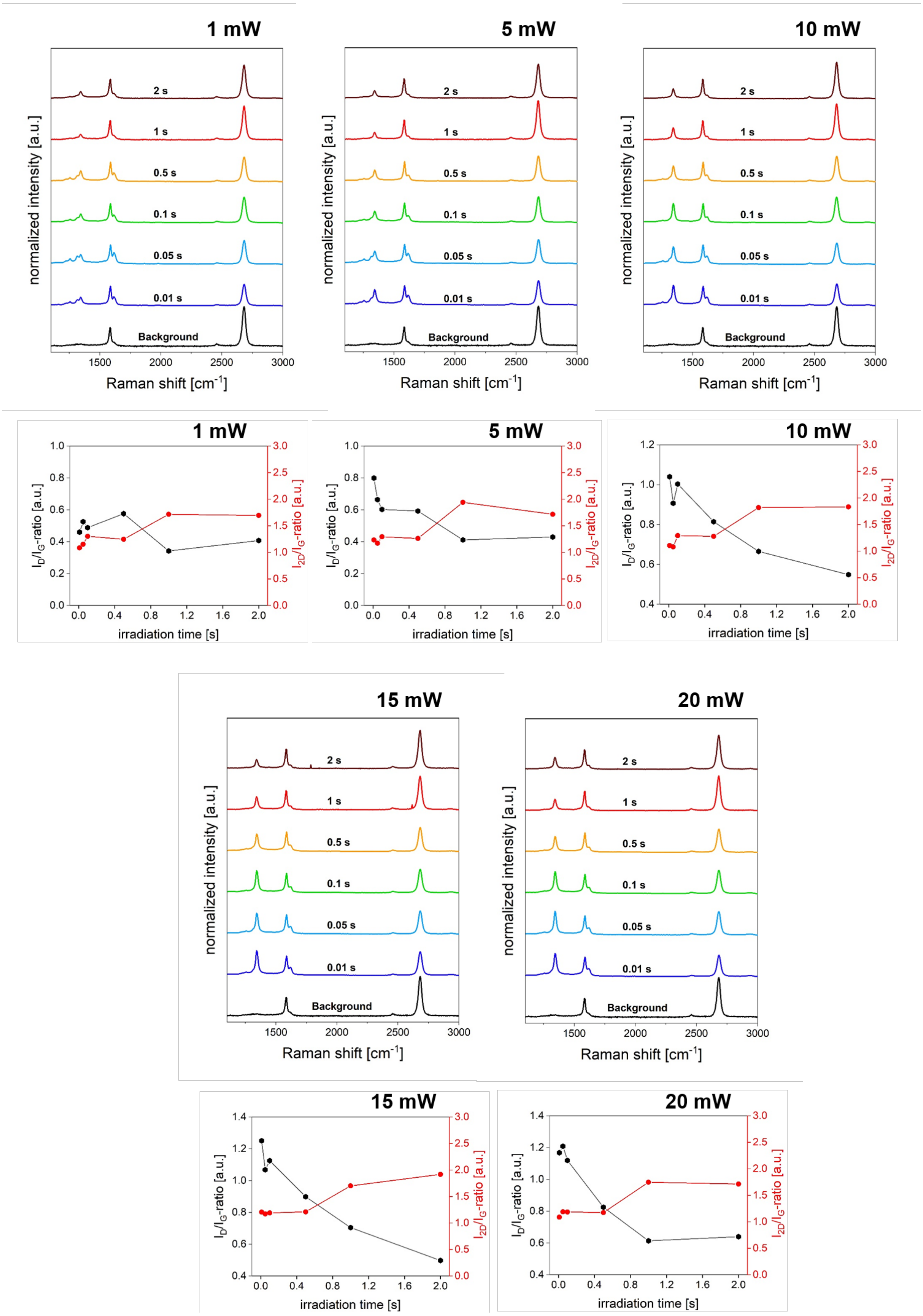

Figure S14: Evolution of the weighted and normalized Raman spectra of laser ‘writing’ with 4-BBD at varying integration times from 0.01 s to 2 s for constant laser powers and the corresponding I_D/I_G and I_{2D}/I_G ratios.

2D Patterning of SLG on Si/SiO$_2$ with 4-methoxybenzenediazonium tetrafluoroborate (4-MBD): SLG on a Si/SiO$_2$ wafer was drop-casted with two drops of 4-MBD in acetonitrile ($2 \cdot 10^{-2}$ M) to create a thick, inhomogeneous coating (see **Fig. S2c**) and the sample was used immediately to avoid decomposition of the compound. ‘Writing’ was performed in the stepwise raster mode. The laser power was varied between 0.01 mW and 10 mW, and the irradiation time was varied between 0.001 s and 5 s. The patterns were written in a stepwise raster mode with 0.5 µm step size and 10 µm spacing between the squares. After ‘writing’, the wafer was immersed in 5 mL acetonitrile for 1 h, sonicated in 5 mL acetone at 37 kHz and 30 % power for 30 s, and washed with a 3 mL IPA/acetone mixture to avoid background functionalization. The ‘readout’ measurement was performed in area scan mode using 4 mW laser power, 0.5 s irradiation time, 1 accumulation, and 0.5 µm step size. **Fig. S15** displays the Raman ‘readout’ map confirming the binding of methoxy-substituted phenyl radicals in the irradiated regions only when applying long irradiation times and low laser powers. Using laser powers below 5 mW, no functionalization of the graphene surface occurred, except for the specific combination of 0.1 mW and 0.01 s. For laser powers above 0.5 mW, in addition with long irradiation times of 2 s and 5 s, the written squares appear blurred, and no defined edges can be resolved in the ‘readout’. The Raman spectra show no characteristic graphene Raman bands in the regions where the lattice has been destroyed (**Fig. S16**).

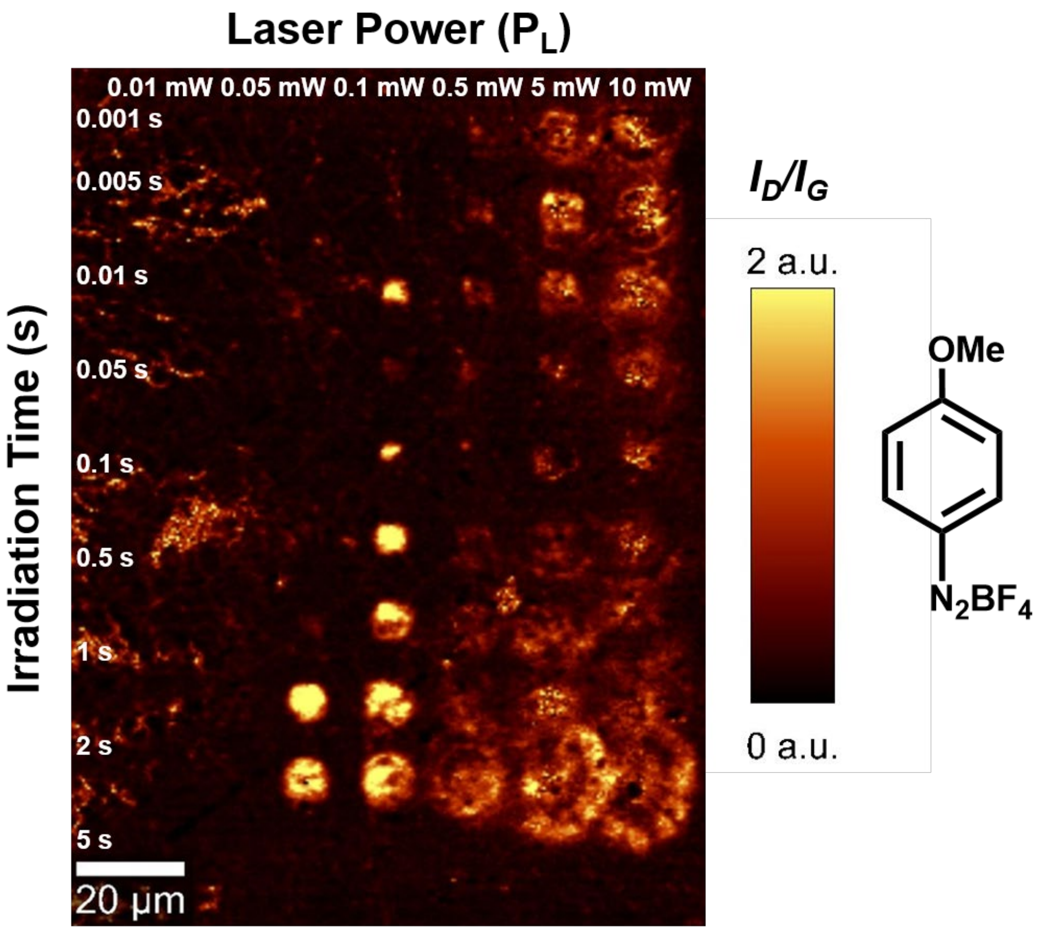

Figure S15: (a) Raman 'readout' map showing the I_D/I_G ratios of the patterned areas with varying laser power from 0.01 mW to 10 mW and increasing irradiation time from 0.001 s to 5 s ('readout' parameters: λ_{exc} = 532 nm, 4 mW laser power, 0.5 s irradiation time, 1 accumulation, and 0.5 µm step size).

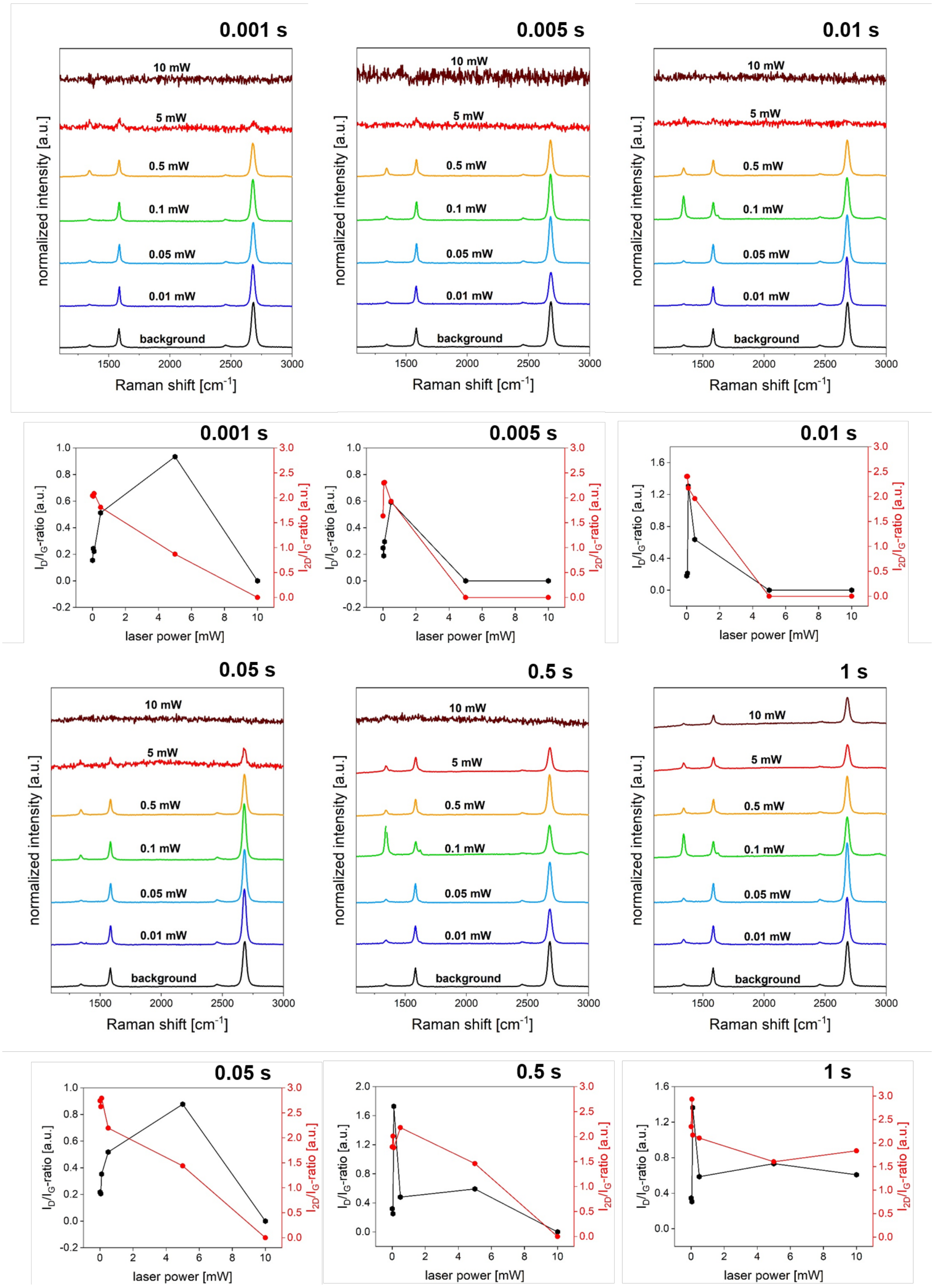
0.001 s
0.005 s
0.01 s
10 mW
5 mW
0.5 mW
0.1 mW
0.05 mW
0.01 mW
background
normalized intensity [a.u.]
Raman shift [cm^{-1}]
I_D/I_G-ratio [a.u.]
I_{2D}/I_G-ratio [a.u.]
laser power [mW]
0.05 s
0.5 s
1 s

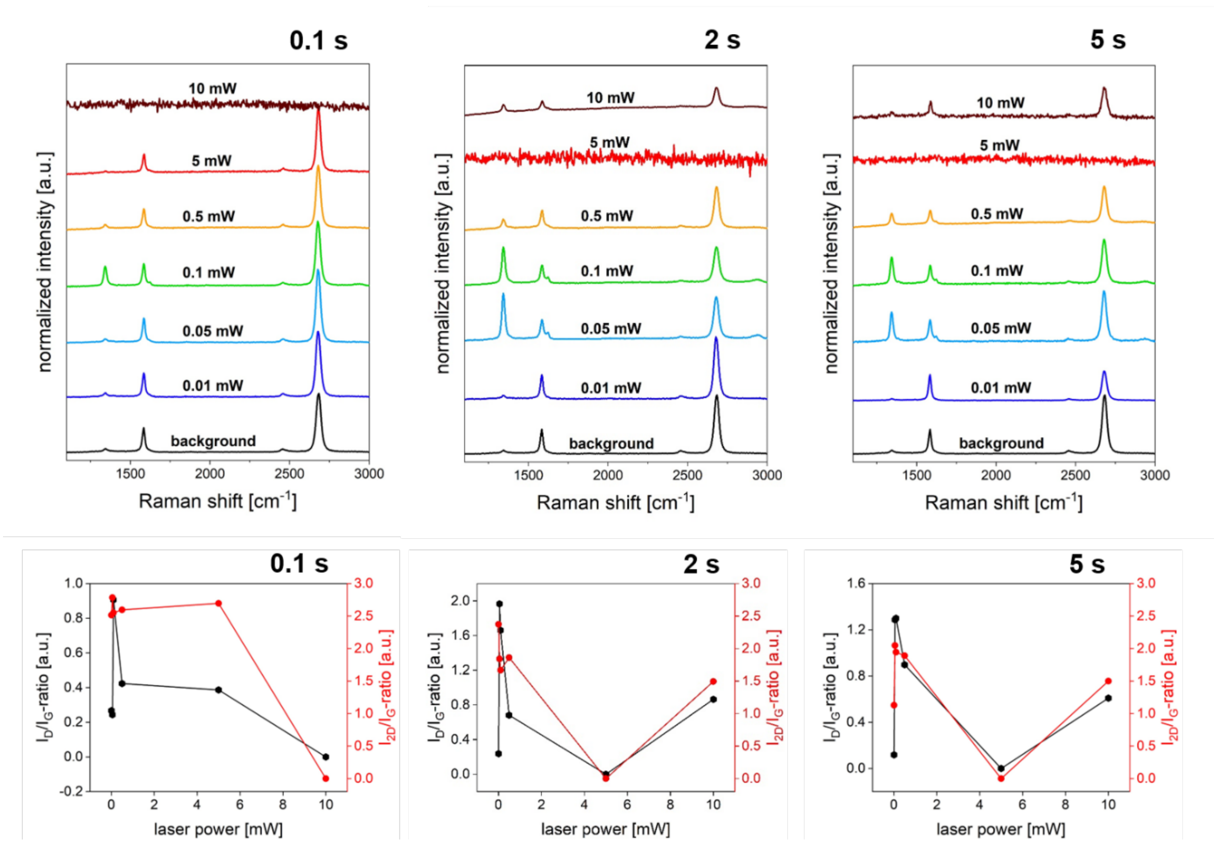

Figure S16: Evolution of the weighted and normalized Raman spectra of laser 'writing' with 4-MBD at varying laser powers from 0.01 mW to 10 mW for constant irradiation times and corresponding I_D/I_G and I_{2D}/I_G ratios.

2.12 Defunctionalization

Temperature-Induced Defunctionalization Studies

For the temperature-induced defunctionalization studies, the temperature control system for microscopy and spectroscopy T96-S, together with a LNP96-S cooling system from Linkam Scientific Instruments was coupled to the Raman setup, described above. The samples were slowly heated up from 20 °C to 300 °C with simultaneous 'readout' measurements using a laser wavelength of 532 nm, a LD 50x Zeiss EC "Epiplan-Neofluar" DIC (NA = 0.5) objective, 10 mW laser power, 0.5 s irradiation time, 1 accumulation, and 0.5 µm step size.

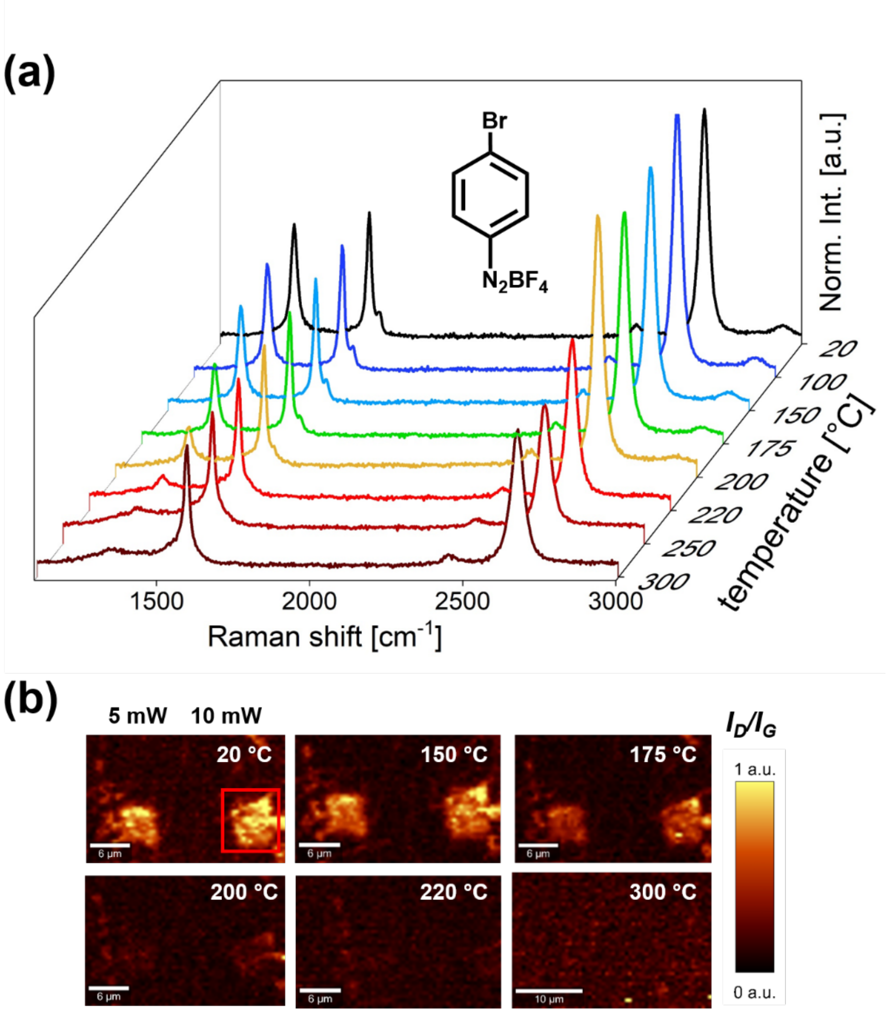

Figure S17: Temperature-dependent Raman study of an area functionalized with 4-BBD. **(a)** Weighted and normalized Raman spectra for different temperatures between 20 °C and 300 °C. **(b)** I_D/I_G ratio maps of the same area (coating: 4-BBD ($2\cdot10^{-3}$ M) and PMMA (0.4 wt %) in acetonitrile, spin-coating, 1 drop, 500 rpm, 'writing' parameters: λ_{exc} = 532 nm, 5 mW-10 mW, 0.05 s, 1 accumulation, 0.5 µm step size, ('readout' parameters: λ_{exc} = 532 nm, 10 mW, 0.5 s, 1 accumulation, 0.5 µm step size).

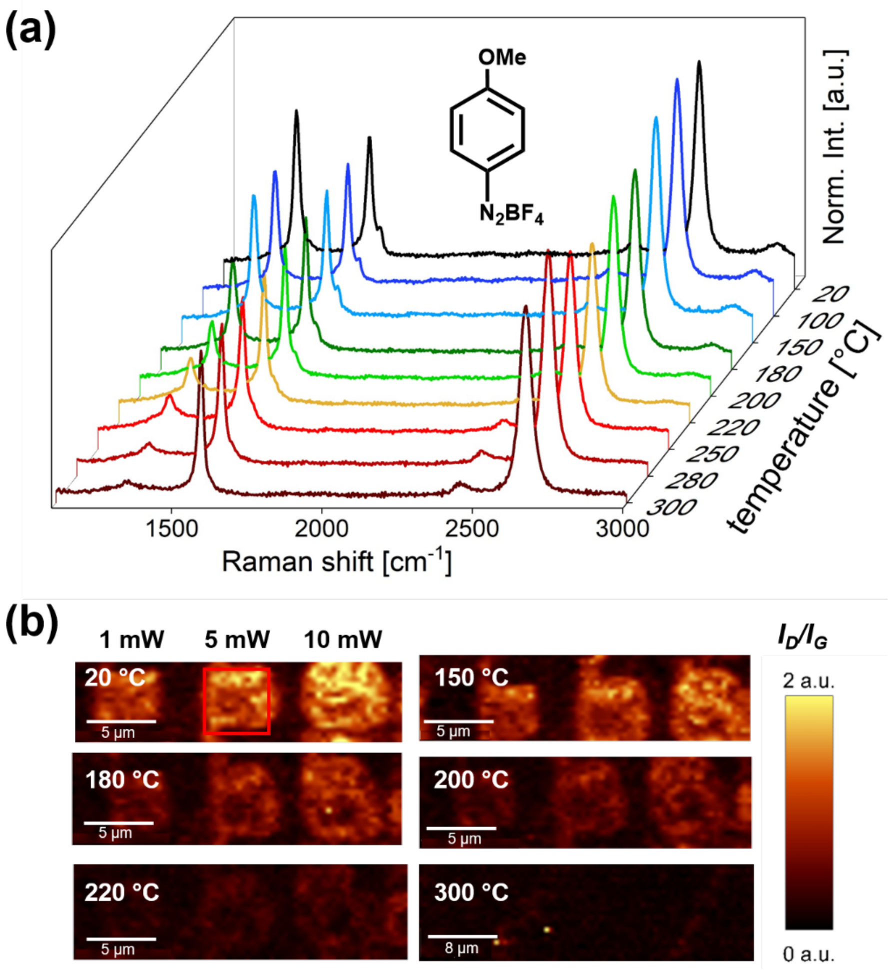

Figure S18: Temperature-dependent Raman study of an area functionalized with 4-MBD. **(a)** Weighted and normalized Raman spectra for different temperatures between 20 °C and 300 °C. **(b)** I_D/I_G ratio maps of the same area (coating: 4-MBD in acetonitrile ($2{\cdot}10^{-2}$ M), drop casting, 2 drops, writing' parameters: λ_{exc} = 532 nm, 1 mW-10 mW, 0.1 s, 1 accumulation, 0.5 µm step size, 'readout' parameters: λ_{exc} = 532 nm, 10 mW, 0.5 s, 1 accumulation, 0.5 µm step size).

Laser-Initiated ‘Erasing’ and ‘Rewriting’

SLG on a Si/SiO_2 wafer was spin-coated with one drop of 4-TBBD in THF ($1\cdot10^{-3}$ M) at 500 rpm for 10 s. The ‘writing’ was performed in area scan mode using 15 mW laser power, 0.05 s irradiation time, 1 accumulation, and 0.5 µm step size. After ‘writing’, the wafer was immersed in 5 mL THF for 30 min and washed with 5 mL THF/IPA and characterized by Raman spectroscopy (‘readout’ parameters: λ_{exc} = 532 nm, 4 mW, 0.3 s, 1 accumulation, 1 µm step size). Laser-based ‘erasing’ was performed in area scan mode using a laser power of 20 mW and varying irradiation times from 10 s to 20 s with 1 accumulation. Characterization was performed by Raman spectroscopy (‘readout’ parameters: λ_{exc} = 532 nm, 4 mW, 0.5 s, 1 accumulation, 0.3 µm step size). For the ‘rewriting’ procedure, a solution of dibenzoyl peroxide (DBPO) in diethyl ether (Et_2O) (c = $1\cdot10^{-3}$ M) was spin-coated with 1 drop of the solution at 4,000 rpm for 2 s. ‘Writing’ was performed on the area of the graphene that was previously treated by the ‘erasing’ procedure using the area scan mode, 0.5 mW laser power, 1 s irradiation time, 1 accumulation, and 0.5 µm step size.[3-5] Afterwards, the sample was immersed in 3 mL Et_2O for 30 min and characterized by Raman spectroscopy (‘readout’ parameters: λ_{exc} = 532 nm, 5 mW, 0.5 s, 1 accumulation, 0.3 µm step size).

3. Further Characterization Methods

AFM and KPFM Images of Areas Functionalized with Aryl Diazonium Salts

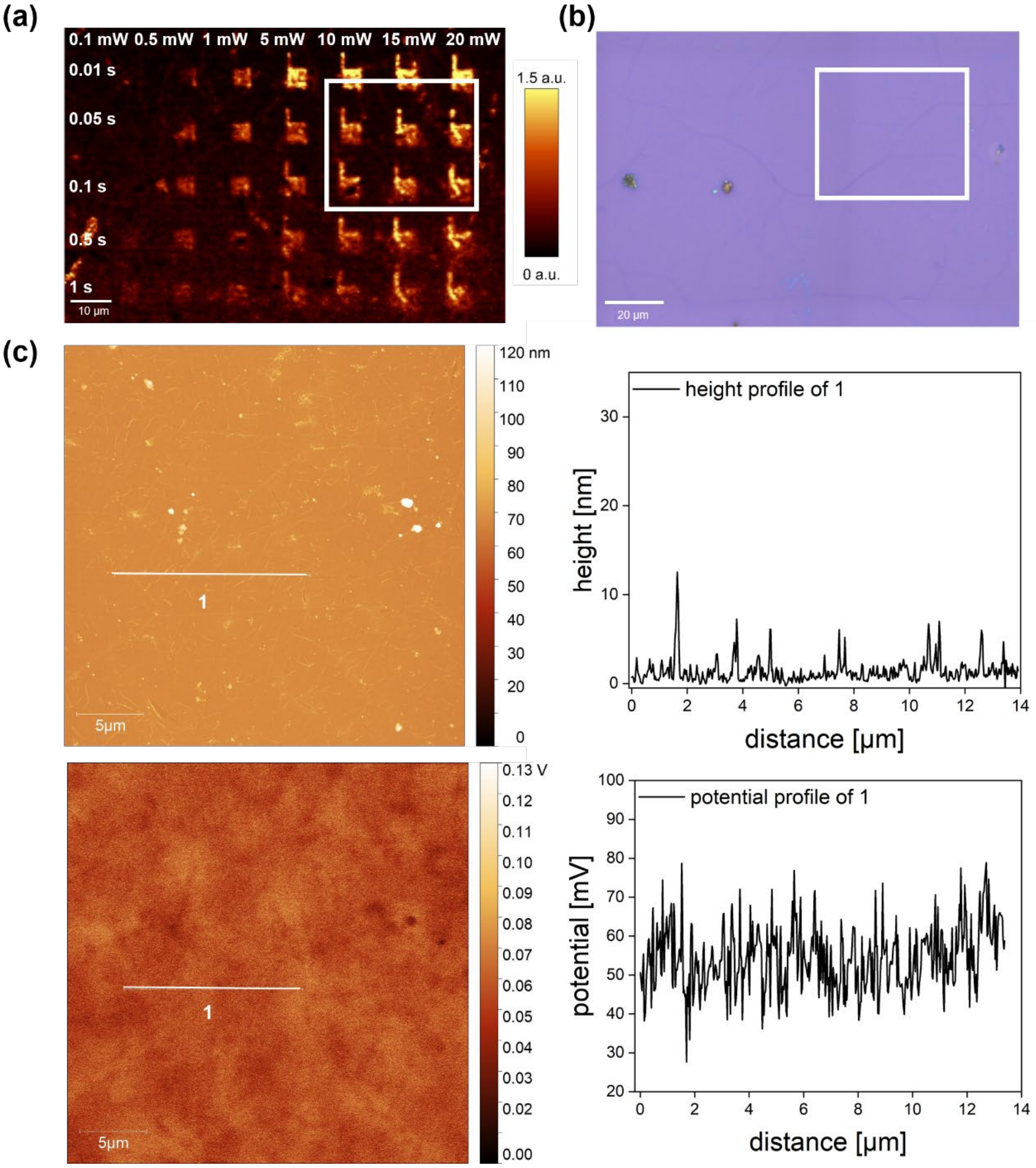

Figure S19: (a) Raman map of the patterned area using 4-TBBD, with the white rectangle indicating the areas of the AFM and KPFM images. **(b)** Optical image (100x magnification) of the patterned regions with the white rectangle indicating the areas of the AFM and KPFM images. **(c)** AFM (top) and KPFM (bottom) images of the functionalized areas with corresponding height and potential profiles (30 x 30 µm scan size, 1024 x 1024 pixel resolution, 0.1 Hz scan rate, 10 µm/s tip velocity).

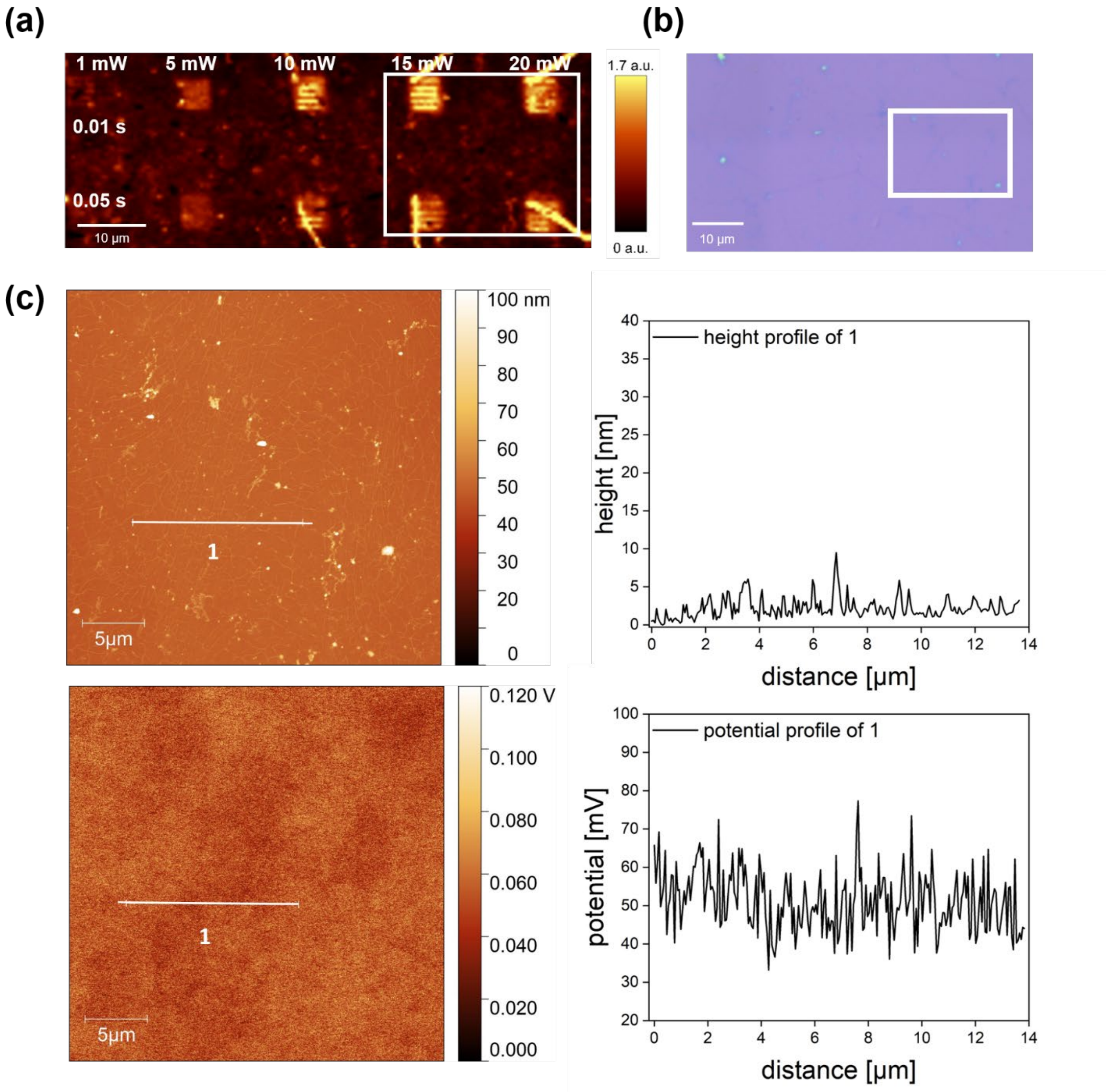

Figure S20: (a) Raman map of the patterned area using 4-BBD, with the white rectangle indicating the areas of the AFM and KPFM images. **(b)** Optical image (100x magnification) of the patterned regions with the white rectangle indicating the areas of the AFM and KPFM images. **(c)** AFM (top) and KPFM (bottom) images of the functionalized areas with corresponding height and potential profiles (30 x 30 µm scan size, 1024 x 1024 pixel resolution, 0.4 Hz scan rate, 10 µm/s tip velocity).

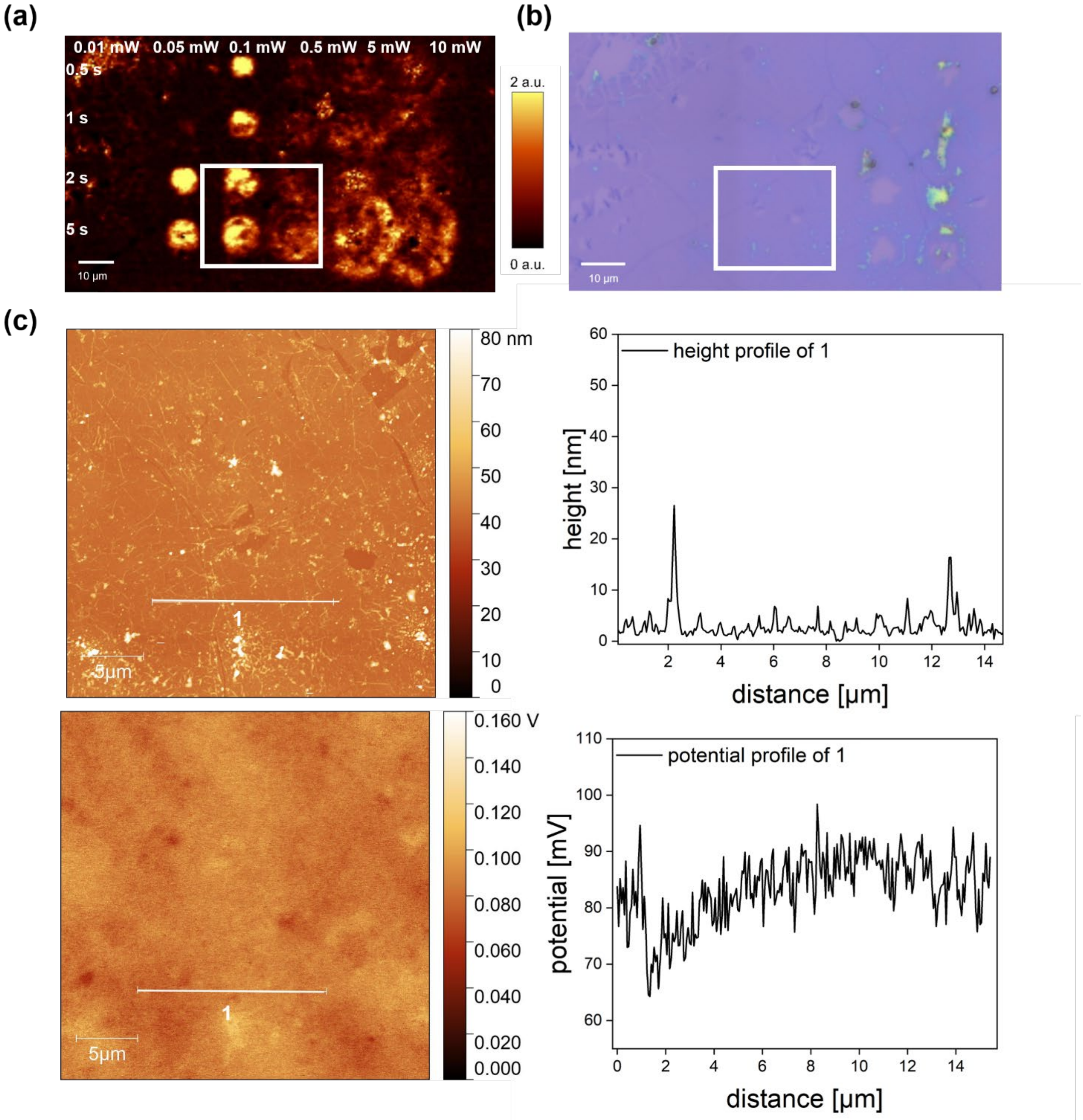

Figure S21: a) Raman map of the patterned area using 4-MBD, with the white rectangle indicating the areas of the AFM and KPFM images. **(b)** Optical image (100x magnification) of the patterned regions with the white rectangle indicating the areas of the AFM and KPFM images. **(c)** AFM (top) and KPFM (bottom) images of the functionalized areas with corresponding height and potential profiles (30 x 30 µm scan size, 512 x 512 pixel resolution, 0.2 Hz scan rate, 10 µm/s tip velocity).

4. Laser ‘Writing’ on Pristine SLG using High Laser Power

As discussed in the paper, high laser power above 5 mW on a highly concentrated layer of 4 MBD leads to partial destruction of the graphene lattice. In order to verify that the high laser power and sonication treatment alone is not responsible for this damage, a reference experiment was performed. For this purpose, a pristine SLG sample on a Si/SiO_2 wafer was irradiated in the area scan mode, 1 accumulation, 0.5 µm step size, with laser powers from 0.1 mW up to 10 mW, while varying the irradiation times from 0.001 s to 5 s. Subsequently, the wafer was immersed in 5 mL of acetonitrile for 1 h and sonicated in 5 mL acetone at 37 kHz and 30 % power for 30 s. The ‘readout’ measurement was performed in area scan mode using 4 mW laser power, 0.5 s irradiation time, 1 accumulation, and 0.5 µm step size. By comparing the optical images of the graphene lattice before (**Fig. S22 left**) and after the laser ‘writing’ process (**Fig. S22 right**), we observed an intact graphene lattice without holes after the laser ‘writing’ and washing process. This result confirms that the hole formation is triggered by the compound and not by the laser ‘writing’ and washing itself.

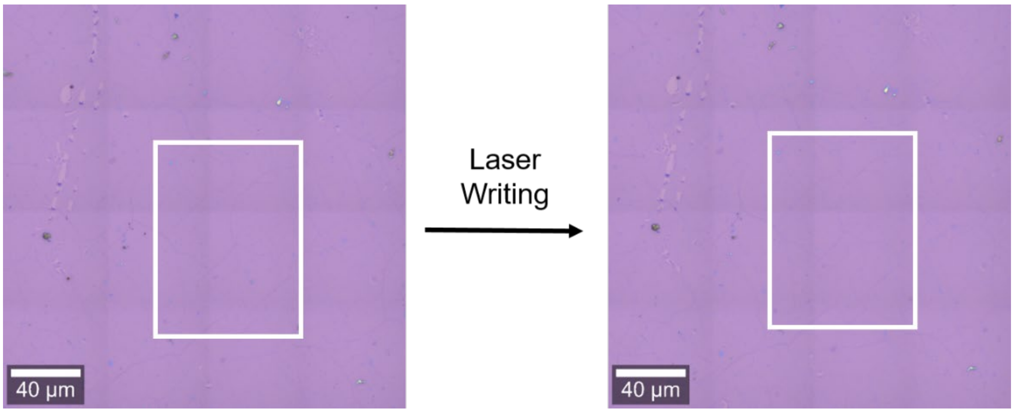

Figure S22: Left: Optical image (100x) of pristine SLG. Right: Optical image (100x) of the same area after laser ‘writing’ with λ_{exc} = 532 nm, 100x objective, area scan mode, laser powers from 0.1 mW to 10 mW, while varying the irradiation times from 0.001 s to 5 s. ‘Readout’ parameters: λ_{exc} = 532 nm, area scan mode, 4 mW laser power, 0.5 s irradiation time, 1 accumulation, and 0.5 µm step size. White rectangle indicates the area of the ‘writing’.

5. Comparison Table: Diazonium Salt/Peroxide/Iodonium Salt

In the following table, we would like to place the present diazonium-based method alongside the group's earlier studies using DBPO and iodonium salt approaches. The data collected for the following table are summarized from this work and the following papers: T. Nagel, S. Wolff, S. Feng, H. Weber, J. Maultzsch, F. Hauke, A. Hirsch,

Carbon **2025**, *241*, 120376 (DBPO) and K. Gerein, D. U. Dzujah, H. Yu, F. Hauke, T. Heine, A. Hirsch, T. Wei, *Angew. Chem. Int. Ed.* **2024**, *63*, e202414090 (iodonium salt).[3, 6]

Table S1: Comparison between three different compound classes for the covalent functionalization of graphene *via* laser writing with regard to various properties.

	**Diazonium Salt** 4-*tert*-butylbenzenediazonium tetrafluoroborate (4-TBBD)	**Peroxide** Dibenzoylperoxide (DBPO)	**Iodonium Salt** Bis(4-fluorophenyl) iodonium triflate
**Radical for functionalization**	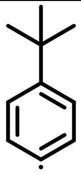	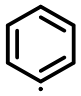	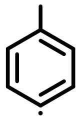
**Electronic effect**	neutral	neutral	electron-withdrawing
**Concentration**	1 mM in THF	1 mM in Et_2O	5 mM in IPA
**Coating method**	spin-coating	spin-coating	spin-coating
**max. I_D/I_G**	~ 2.6 (532 nm, 0.8 mW, 60 s)	~ 3.1 (532 nm, 0.5 mW, 2 s)	~ 2.5 (532 nm, 12 mW, 1 s)
**min. I_{2D}/I_G**	~ 0.78 (532 nm, 0.8 mW, 60 s)	~ 0.38 (532 nm, 0.5 mW, 5 s)	~ 0.70 (532 nm, 13 mW, 1 s)
**Resolution**	needs to be further determined; probably transferable	~ 1.5 µm	~ 2 µm
**Excitation wavelength**	457 nm **<** 532 nm **<** 633 nm	457 nm **>** 532 nm **>** 633 nm	457 nm **>** 532 nm **>** 633 nm
**Absorption bands in UV-Vis**	No absorption in visible region	No absorption in visible region	No absorption in visible region
**Proposed mechanism**	hot-electron transfer into functionalization reagent	hot-electron transfer into functionalization reagent	hot-electron transfer into functionalization reagent
**Defunctionalization**	thermal Δ **and** via h*v*	thermal Δ **and** via h*v*	thermal Δ **and** via h*v*

6. References

[1] K. F. Edelthalhammer, D. Dasler, L. Jurkiewicz, T. Nagel, S. Al-Fogra, F. Hauke, A. Hirsch, *Angew. Chem. Int. Ed.* **2020**, *59*, 23329-23334.

[2] R. Sharma, J. H. Baik, C. J. Perera, M. S. Strano, *Nano Lett.* **2010**, *10*, 398-405.

[3] T. Nagel, S. Wolff, S. Feng, H. Weber, J. Maultzsch, F. Hauke, A. Hirsch, *Carbon* **2025**, *241*, 120376.

[4] T. Nagel, K. Gerein, F. Hauke, A. Hirsch, *Adv. Sci.* **2025**, e11481.

[5] T. Nagel, F. Hauke, A. Hirsch, *manuscript in preparation*.

[6] K. Gerein, D. U. Dzujah, H. Yu, F. Hauke, T. Heine, A. Hirsch, T. Wei, *Angew. Chem. Int. Ed.* **2024**, *63*, e202414090.
